# Single Cell Based Phosphorylation Profiling Identifies Alterations in Toll-Like Receptor 7 and 9 Signaling in Patients With Primary Sjögren's Syndrome

**DOI:** 10.3389/fimmu.2019.00281

**Published:** 2019-02-21

**Authors:** Richard Davies, Irene Sarkar, Daniel Hammenfors, Brith Bergum, Petra Vogelsang, Silje M. Solberg, Sonia Gavasso, Johan G. Brun, Roland Jonsson, Silke Appel

**Affiliations:** ^1^Broegelmann Research Laboratory, Department of Clinical Science, University of Bergen, Bergen, Norway; ^2^Department of Rheumatology, Haukeland University Hospital, Bergen, Norway; ^3^Department of Dermatology, Haukeland University Hospital, Bergen, Norway; ^4^Department of Clinical Science, University of Bergen, Bergen, Norway

**Keywords:** Sjögren's syndrome, extraglandular manifestations, autoantibodies, phosphoflow, Toll-like receptors, type I interferon

## Abstract

Primary Sjögren's syndrome (pSS) is associated with polymorphisms and mRNA expression profiles that are indicative of an exaggerated innate and type I IFN immune response. Excessive activation potential of signaling pathways may play a role in this profile, but the intracellular signaling profile of the disease is not well characterized. To gain insights into potentially dysfunctional intracellular signaling profiles of pSS patients we conducted an exploratory analysis of MAPK/ERK and JAK/STAT signaling networks in peripheral blood mononuclear cells (PBMC) from 25 female pSS patients and 25 female age-matched healthy donors using phospho-specific flow cytometry. We analyzed unstimulated samples, as well as samples during a 4 h time period following activation of Toll-like receptor (TLR) 7 and 9. Expression levels of *MxA, IFI44, OAS1, GBP1*, and *GBP2* in PBMC were analyzed by real-time PCR. Cytokine levels in plasma were determined using a 25-plex Luminex-assay. Principal component analysis (PCA) showed that basal phosphorylation profiles could be used to differentiate pSS patients from healthy donor samples by stronger intracellular signaling pathway activation in NK and T cells relative to B cells. Stimulation of PBMC with TLR7 and −9 ligands showed significant differences in the phosphorylation profiles between samples from pSS patients and healthy donors. Including clinical parameters such as extraglandular manifestations (EGM), we observed stronger responses of NF-κB and STAT3 S727 in B cells from EGM-negative patients compared to EGM-positive patients and healthy controls. Plasma cytokine levels were correlated to the basal phosphorylation levels in these patients. In addition, 70% of the patients had a positive IFN score. These patients differed from the IFN score negative patients regarding their phosphorylation profiles and their plasma cytokine levels. In conclusion, we here report increased signaling potentials in peripheral B cells of pSS patients in response to TLR7 and −9 stimulation through STAT3 S727 and NF-κB that correlate with a type I IFN signature. Induction of these pathways could contribute to the generation of a type I IFN signature in pSS. Patients displaying elevated potentiation of STAT3 S727 and NF-κB signaling could therefore benefit from therapies targeting these pathways.

## Introduction

Sjögren's syndrome (SS) is a systemic autoimmune disease characterized by lymphocytic infiltrates of the salivary and lacrimal glands. The hallmarks of the disease are dryness of the mouth (xerostomia) and the eyes (keratoconjunctivitis sicca) (1, 2). This dryness and other clinical manifestations result in a significant decrease in quality of life. Currently there is no cure or effective disease modifying treatment for SS, with management of the disease based on the relief of symptoms. The lack of effective treatments is linked to the pathogenic complexity of the disease, with genetic predisposition, hormonal, and environmental factors all contributing to disease etiology and pathogenesis. While almost all SS patients display abnormal tear and/or saliva secretion (3), there is significant heterogeneity in the disease manifestations, pathology and clinical course. This heterogeneity may reflect distinct patient subgroups with unique pathophysiologic mechanisms (4). For example, Sjögren's syndrome can present with a wide range of extraglandular manifestations (EGM) including fatigue and constitutional, musculoskeletal, articular, cutaneous, pulmonary, liver, and kidney involvement, as well as neuropathies and lymphomas (5). B cell hyperactivity is also a common feature of SS. It can manifest as hypergammaglobulinaemia and presence of autoantibodies including anti-Sjögren's syndrome A (SSA) and anti-Sjögren's syndrome B (SSB) (5) often preceding clinical symptoms (6).

Aspects of SS pathogenesis that have gained considerable attention during recent years are abnormal cytokine production and genetic associations. Of prominent interest are features associated with type I interferon (IFN). The type I IFN family consists of multiple members including IFN-α and β, and they are involved in various biological functions including defense against viral or bacterial infection, immune-modulation, and negative regulation of proliferation (7). An activated type I IFN system known as the interferon signature plays an important role in different autoimmune diseases, amongst them pSS (7, 8). In pSS patients, the interferon signature is associated with higher disease activity index scores (9).

It has been speculated that the initiating factor in the activated type I IFN response is a genetically determined exaggerated innate immune response against inappropriately overexpressed endogenous or exogenous danger signals. Extracellular nucleic acids present during viral infections, for example, can induce type I IFN production through interactions of extracellular nucleic acid with endosomal receptors, including TLR3, TLR7, and TLR9 (7). In SS it has been speculated that expression of danger signals resulting from transient or persistent viral infection of epithelial cells leads to continuous activation of TLR signaling eventually contributing to SS pathogenesis (7). Interestingly, a number of infectious agents including Epstein-Barr virus, human T-lymphotropic virus type 1, hepatitis C virus and enterovirus have been reported as potential initiators of glandular lesions in SS patients (7).

Dysfunctional intracellular signaling mechanisms may influence the immunological response of a cell to a given stimulus, affecting transduction of a given signal and resulting in aberrant gene expression. We have previously shown that patients with pSS have an altered response of PBMC to IFN stimulation (10). Interestingly, several genetic variants associated with SS function in downstream signaling from TLRs or their regulation, including *IRF5* (11, 12), *IL-10* (13), *I*κ*B*α (14), TNFAIP3 interacting protein 1 (*TNIP1*) (12), and *OAS1* (15). Potentiation, chronic activation or dysregulation of TLR signaling pathways could lead to exaggerated production of type I IFN and contribute to the type I IFN signature and disease pathogenesis. However, not much is known about TLR signaling in patients with pSS.

In this study, we characterized intracellular signaling pathways including those downstream from TLR7 and −9 receptor activation in PBMC by phospho-specific flow cytometry (phosphoflow) (16). We focused here on direct targets of TLR signaling such as ERK/MAPK as well as epitopes activated upon IFN signaling such as JNK/STAT. Increased induction of phosphorylation of STAT3 S727 and NF-κB was observed in B cells from pSS patients following TLR7 and −9 stimulation compared to B cells from healthy donors. The activation was shown to be increased in patients with SSA autoantibodies and patients without extraglandular manifestation. The increased responses following TLR7 and −9 stimulation through STAT3 S727 and NF-κB in B cells were associated with increased expression of three genes upregulated in response to type I IFN (*MxA, IFI44, OAS1*) but not type II IFN inducible genes (*GBP1* and *GBP2*). Plasma cytokine levels were different in SSA+ and SSA– patients and correlated with basal phosphorylation levels of several phospho-epitopes in patient subgroups. In conclusion, this study provides support that enhanced responses through TLR7 and −9 may play a role in the induction of a type I IFN signature observed in pSS patients indicating viral infections as potential trigger of the disease. Alternatively, induced expression of type I IFN inducible genes may potentiate TLR7 and −9 responses. Patients displaying elevated potentiation of these pathways may therefore benefit from therapies targeting these pathways.

## Materials and Methods

### Blood Sampling

Peripheral blood from patients with pSS was collected in Lithium-heparin tubes (BD diagnostics) at the Department of Rheumatology, Haukeland University Hospital, Bergen, Norway. Blood from healthy age- and gender-matched donors was collected at the blood bank at the Haukeland University Hospital in Bergen, Norway. PBMC were isolated by density gradient centrifugation with lymphoprep^TM^ (Axis-Shield, Oslo, Norway) and cryopreserved as described previously (17). Plasma was aliquoted and stored at −70°C, and PBMC were stored at −150°C for ~12–16 months. All patients fulfilled the pSS American-European Consensus group (AECG) criteria (18) and displayed no additional autoimmune diseases or lymphoma. An overview of the cohort is shown in Table 1. The study was approved by the regional ethical committee (#2009/686). All participants provided written informed consent.

**Table 1:** Characteristics of patients and controls used in study.

	**Sjögren's syndrome**	**Healthy controls**
**COHORT CHARACTERISTICS**
Females/males	25/0	25/0
Age, median (range) years	56 (33–73)	54 (42–70)
**CLINICAL FEATURES (PATIENTS)**
SSA antibodies (%)	19 (76)	
SSB antibodies (%)	12 (48)	
SSA and SSB antibodies (%)	12 (48)	
ANA (%)	19 (76)	
Positive Schirmer's test (tear flow < 5 mm/5 min) (%); *n* = 24	14 (58.3)	
Focus score^†^ ≥ 1 (%); *n* = 14	10 (71.4)	
ESR, high levels^††^	5 (20)	
CRP high levels (≥5 mg/L)	2 (8)	
Extraglandular manifestations (%)	14 (56)	
Treatment		
DMARDs	8 (32)	
Corticosteroids	2 (8)	

*Continuous data is expressed as median. Categorical data is expressed as frequency and percentage*.

† *Focus score indicates the number of inflammatory foci containing more than 50 mononuclear cells per 4 mm^2^ biopsy tissue*;

†† *Age and gender dependent. DMARDs, disease-modifying anti-rheumatic drugs; ANA, anti-nuclear antibodies; ESR, erythrocyte sedimentation rate; CRP, C-reactive protein*.

### Routine Laboratory Assays

Identification of anti-Ro/SSA and anti-La/SSB, other antinuclear antibodies (ANA), erythrocyte sedimentation rate (ESR), C-reactive protein (CRP), and extraglandular manifestations were obtained as part of routine clinical investigation at time of blood sampling. SSA, SSB, and ANA were reported as either present or absent, while other serum and blood parameters were reported as continuous values. Extraglandular manifestations were defined as disease features outside surface exocrine glands.

### Real-Time Quantitative PCR

Total RNA was isolated from PBMC of 20 pSS patients and 17 healthy controls and transcribed into cDNA as described previously (10). The following Taqman gene expression assays were utilized: Hs00895608_m1 (*MxA*); Hs00973637_m1 (*OAS1*); Hs00951349_m1 (*IFI44*); Hs00977005_m1 (*GBP1*); Hs00894837_m1 (*GBP2*); Hs03928990_g1 (18S rRNA) (all Thermo Fisher Scientific, Waltham, USA). All PCR reactions were run in duplicates on a Light Cycler 480 (Roche Diagnostics, Oslo, Norway). 18S rRNA was used as reference gene, and relative expression levels were calculated as 2^−Δ*Ct*^. The IFN score was calculated according to Feng et al. (19) by standardizing expression levels using mean and SD of the healthy controls for the respective gene and using the following formula:

$$\begin{array}{l} \overset{\text{3}}{\underset{i}{\sum }}\;=\frac{gene\;i_{pSS}-mean\;gene\;i_{Ctr}}{SD\;\left(gene\;i_{Ctr}\right)} \end{array}$$

where *i* = each of the 3 type I IFN-inducible genes (*MxA, IFI44, OAS1*), *gene i*_*pSS*_ = the gene expression level in each pSS patient, and *gene i*_*Ctr*_ = the gene expression in controls. To set a threshold, 3 × SD of healthy controls was utilized.

### Antibodies Used for Flow Cytometry

The following phospho-specific monoclonal antibodies were used in 3 different panels during the flow cytometry protocol described previously (17): Alexa Fluor®647 conjugated anti-STAT4 (pY693, clone 38/p-STAT4, panel 1), anti-STAT 1 (pS727, clone K51-856, panel 2), and anti-STAT3 (pS727, clone 49/p-STAT3, panel 3); PerCP-Cy^TM^ 5.5 conjugated anti-ERK1/2 (pT202/pY204, clone 20A, panel 1), anti-STAT1 (pY701, clone 4a, panel 2), and anti-STAT3 (pY705, clone 4/P-STAT3, panel 3); and PE-Cy^TM^7 conjugated anti-p38 MAPK (pT180/pY182, clone 36/p38, panel 2), and anti NF-κB p65 (pS529, clone K10-895.12.50, panel 1), anti-STAT5 (pY694, clone 47/STAT5(pY694), panel 3) (all from BD Biosciences, San Jose, CA, USA). Cell surface markers incorporated in the assays were BV786 conjugated anti-CD3 (clone SK7, BD Horizon^TM^), Alexa Fluor® 488 conjugated anti-CD20 (clone H1 (FB1), BD Biosciences) and PE conjugated anti-CD56 (clone N901, Beckmann Coulter, CA, USA).

### Cell Culture and Stimulation

Before stimulation, cryopreserved PBMC were rapidly thawed using a water bath set to 37°C and washed once in prewarmed X-vivo 20^TM^ by centrifugation at 300 g for 7 min. The cells were then resuspended in prewarmed X-vivo 20^TM^ and rested at 37°C at 5% CO_2_ for 30 min before the cell concentration was adjusted to 3 × 10^6^ cells/ml in X-vivo 20^TM^. Two hundred microliters were dispensed into 7 wells of a Megablock® 96 well plate (Starstedt, Nümbrecht, Germany), along with 2 wells of a reference sample. The cells were rested at 37°C with 5% CO_2_ for 2 h. Following, the cells were either left unstimulated or stimulated according to a reverse time course for 15, 30, 60, 120, 180, or 240 min with a combination of TLR7 (CL097; Invivogen) and −9 ligands (CpG type B ODN 2006 and type C ODN 2395; Invivogen, Carlsbad, California, USA) at 2 μg/ml each. Due to limited cell numbers and samples, time points were excluded in 2 patients, both for 60 and 120 min, and 1 healthy control for 180 and 240 min.

### Fluorescent Cell Barcoding and Phospho-Epitope Staining for Flow Cytometry

PBMC were fixed by adding 16% PFA (Electron Microscopy Sciences (Hatfield, PA, USA) warmed to 37°C directly into the PBMC cultures resulting in a final PFA concentration of 1.5%. The samples were mixed thoroughly by pipetting. The cells were fixed at RT for 10 min before pelleting at 1,000 g for 5 min. The PBMC were then vigorously resuspended by vortexing in 50 μl PBS before drop wise addition of 1 ml ice cold methanol and incubation on ice for 30 min. The permeabilized cells were kept overnight at −80°C. After washing with PBS, the PBMC were stained according to a 3 × 3 barcoding grid (9 stimulation conditions) using 3 levels of pacific orange (PO) and pacific blue (PB) succinimidyl ester dyes (PB 100, 25, and 6.3 ng/ml; PO 250, 70, and 0 ng/ml; Life Technologies, Grand Island, NY, USA) for 30 min in the dark at 4°C in a volume of 1 ml. Barcoded PBMC were then washed once with staining media (PBS containing 1% BSA), and the 9 different dye concentration/combination samples were combined into one sample. The sample was washed and incubated with 2 μl Fc receptor block (Miltenyi Biotec, Bergisch Gladbach, Germany) per 1 × 10^6^ cells for 10 min on ice. Following, the sample was subdivided into 3 parts and incubated for 30 min at RT in the dark with the 3 different antibody staining panels. An aliquot of the barcoded cells was collected before addition of antibody as a barcoding only control. The samples were then washed twice and re-suspended in staining medium containing 2 mM EDTA (Sigma-Aldrich) prior to analysis.

### Flow Cytometry Data Analysis

Samples were acquired on a LSRI Fortessa flow cytometer (BD Biosciences, San Jose, CA, USA) with BDFACSDiVa™ Software (BD Biosciences) at the Bergen Flow Cytometry Core Facility, University of Bergen, Norway. The flow cytometer was equipped with 407, 488, 561, and 635 nm lasers, and emission filters for PerCP-Cy5.5 (LP: 685, BP: 695/40), Alexa Fluor-488 (LP: 505, BP: 530/30), PE-Cy7 (LP: 750, BP: 780/60), PE (LP: –, BP: 582/15), APC (LP: –, BP: 670–/-14), Pacific blue (LP: –, BP:450/50), Pacific orange (LP: 570, BP: 585/42), and BV 786 (LP: 750, BP: 780/60). The cytometer was routinely calibrated with BD cytometer setup and tracking beads (BD Biosciences). A minimum of 200,000 events in the intact cell gate was collected for each sample, giving a minimum of 2,000 events per analyzed cell population (T cells, B cells, NK cells). Flow cytometry data were analyzed in FlowJo (Tree Star) and Cytobank (http://www.cytobank.org). A representative gating strategy and phosphorylation profile for a single donor is shown in Supplementary Figure S1. Cryopreserved PBMC from a single donor with unstimulated and stimulated samples were run in each assay as a positive control for inter-assay normalization and assessing assay to assay variability. Median fluorescence intensities (MdFI) for gated populations were exported to Microsoft excel. The raw flow cytometry data for which this article is based can be found at the flow data repository of the International Society for Advancement of Cytometry (20), FR-FCM-ZYED. The robustness of the flow cytometry assay used was previously established and published, see “An optimized multiplex flow cytometry protocol for the analysis of intracellular signaling in peripheral blood mononuclear cells” (17). Relevant information for repeating the experiment as presented in “The minimum information about a Flow Cytometry Experiment (MIFlowCyt)” (21) are provided in Supplementary Table S1.

### Cytokine Determination

Cytokine and chemokine concentrations were determined in plasma samples using a 25-plex Luminex assay cytokine and chemokine panel (Invitrogen, catalog number LHC0009M) and run on a Luminex 100 System (Luminex Corporation, Austin, TX) according to the manufacturer's instructions.

### Statistical Analysis

Generation of graphs and comparisons between categories were done using an Unpaired Mann-Whitney test using Graphpad Prism (version 6.05). Differences were considered statistically significant when *p* ≤ 0.05. The analysis was exploratory in nature hence no correction was made for multiple comparisons. Principle component analysis (PCA) using Unscrambler® X software (Camo software) was used to reduce dimensionality of the dataset and find clusters of patients with similar signaling profile which could be used to differentiate between disease status, presence of SSA autoantibodies, EGM and medication (DMARDs and corticosteroids). PCA was performed using the algorithm NIPALS, the data was mean centered and run with no weighting for change of MdFI, and weighted for absolute MdFI by dividing by standard deviation. Two methods were used to remove “redundant” variables to simplify interpretation and focusing subsequent analysis. First variables that described <50% of the variation were removed from the initial PCA, than if appropriate stepwise reduction of less significant variables with low variable leverage was performed. Correlations were assessed by the Spearman's rank test, with outliers removed using robust regression and outlier removal (ROUT) method and a ROUT coefficient Q of 1 used.

Since most of the cytokine data did not follow a normal distribution, Mann-Whitney U test was performed to study significant differences between the groups, and Spearman's correlation was used to find any significant relationships between the cytokines and the phosphoproteins. Degree of correlation was determined according to the recommendation of the British Journal of Medicine (https://www.bmj.com/about-bmj/resources-readers/publications/statistics-square-one/11-correlation-and-regression)—*r* = 0.4–0.59 (moderate), *r* = 0.6–0.79 (strong), and *r* = 0.8–1.0 (very strong). Analysis was done using GraphPad Prism 7 and *p* ≤ 0.05 was considered to be statistically significant.

## Results

### PBMC From pSS Patients Display Shifts in Phosphorylation States of Proteins Involved in Signaling Pathways

In order to reveal possible dysfunctional intracellular signaling mechanisms upon TLR stimulation in pSS patients, we here analyzed MAPK/ERK and JAK/STAT signaling networks in peripheral blood cells from female pSS patients and female age-matched healthy donors in unstimulated cells and upon stimulation with a combination of TLR7 and −9 ligands. In this pilot study, we limited our analyses to the main lymphocyte populations (T cells, B cells, NK cells) as all have been shown to be affected by TLR7/9 stimulation (22–24). An overview of unstimulated and TLR stimulated measurements can be found in the Supplementary Table S2 (T cells), Supplementary Table S3 (B cells), and Supplementary Table S4 (NK cells).

**Figure 1:**
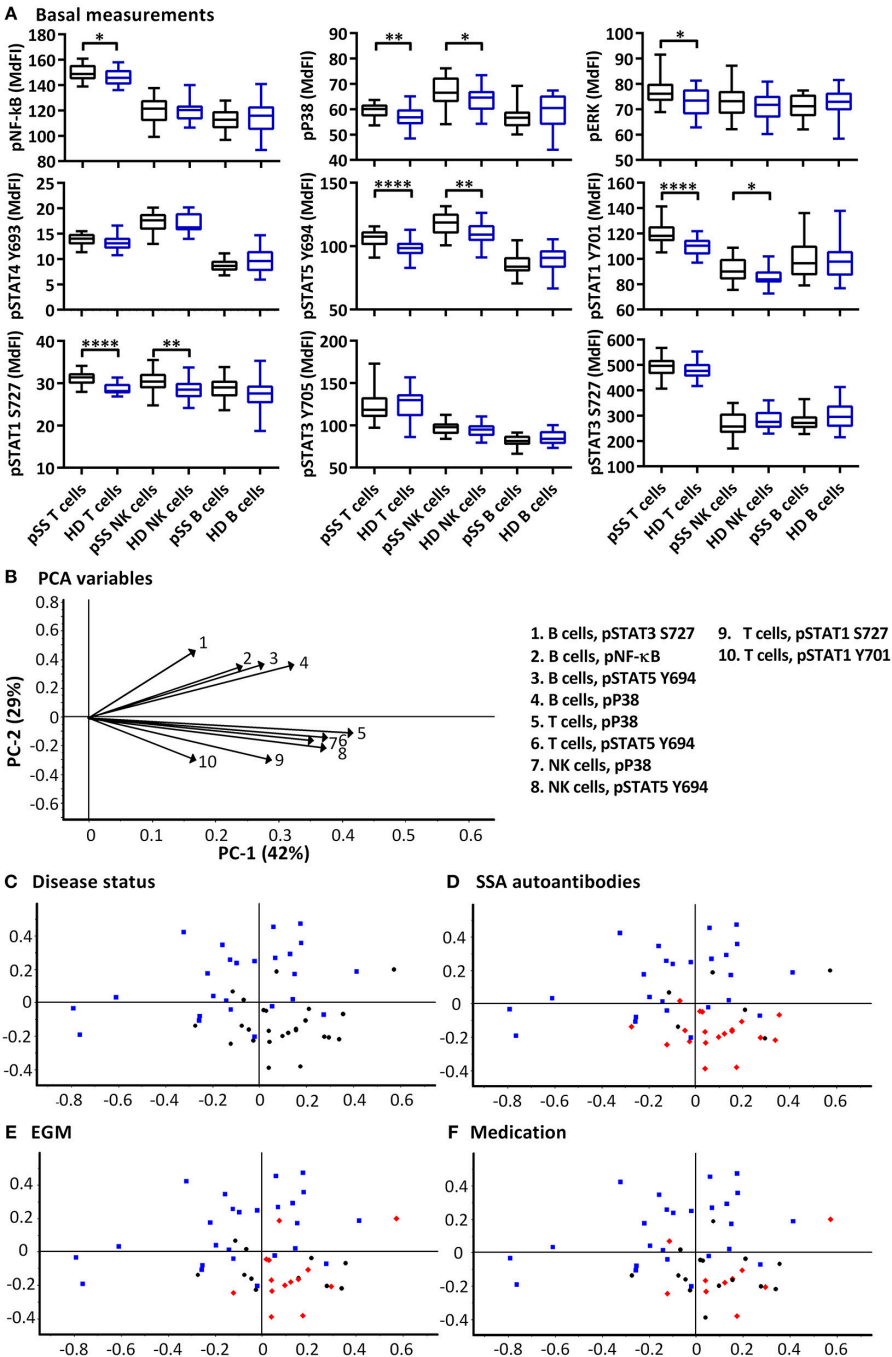
Basal phosphorylation profiles in B cells, T cells and NK cells of pSS patients differ compared to healthy controls. Basal phosphorylation levels of NF-κB, P38, ERK1/2, STAT4 Y693, STAT5 Y694, STAT1 Y701, STAT1 S727, STAT3 Y705, and STAT3 S727 were analyzed by flow cytometry in T cells, B cells and NK cells are given in **(A)**. Comparisons of phosphorylation levels (MdFI) between healthy donor (blue) and pSS patient (black). Comparisons between pairs were done using an Unpaired Mann-Whitney test. Graphs show the median, 25–75 percentiles and minimum and maximum. Differences were considered statistically significant when *p* ≤ 0.05, with significance indicated as * ≤ 0.05, ** ≤ 0.01, and **** ≤ 0.0001. PCA analysis of the profiles is given in **(B–F)**, disease status is highlighted in **(C)**, SSA autoantibody positivity in **(D)**, presence of EGM in **(E)**, and medication (DMARDs or corticosteroids) use in **(F)**, with healthy donors, blue squares; pSS patients, black circles; SSA+, EGM+, or medicated patients, red diamonds (**D–F**, respectively). The loading plot, which contains information about the variable for the corresponding PCA is given in **(B)**, with variable indicated by vectors and the key to the right. Variables contributing little to the PCA are plotted around the center as denoted by the gray axis, while variables that have high contributions are plotted further from the axes. After initial calculation of principal components, the model was recalculated with only variable explaining >50% of the variance retained. The data represents 25 healthy controls and 25 patients pooled from 13 independent experiments.

Significant increases of basal phosphorylation in cells from pSS patients were observed in T cells for NF-κB, P38, ERK, STAT5, STAT1 Y701, STAT1 S727, and NK cells for P38, STAT5, STAT1 Y701, and STAT1 S727 compared to healthy donors. B cells showed no significant differences in basal phosphorylation (Figure 1A).

PCA was used to concurrently relate multiple basal signaling states to various clinical parameters such as production of SSA autoantibodies, presence of extraglandular manifestations (EGM) and medication (DMARDs and corticosteroids) within the patient cohort (Figures 1B–F). Using basal phosphorylation levels, pSS patients could be separated from healthy donors (Figure 1C). Spatial groupings indicated closer similarities within the pSS and healthy donor cohorts than between the groups. Separation of pSS and healthy donor samples was primarily along PC2 which explained 29% of the variation. Examination of the loading plot (Figure 1B) indicated differences between basal signaling phenotype of pSS patients and healthy donors, with pSS patients showing weaker basal pathway activation in B cells relative to NK and T cells compared to healthy donor cells. No groupings were shown along PC1 which explained 42% of the variation. Including clinical parameters in the analysis, patients without autoantibodies against SSA grouped closer to the healthy controls (Figure 1D), while patients with EGM (Figure 1E), and patients prescribed DMARDs or corticosteroids (Figure 1F) grouped throughout the pSS cluster.

**Figure 2:**
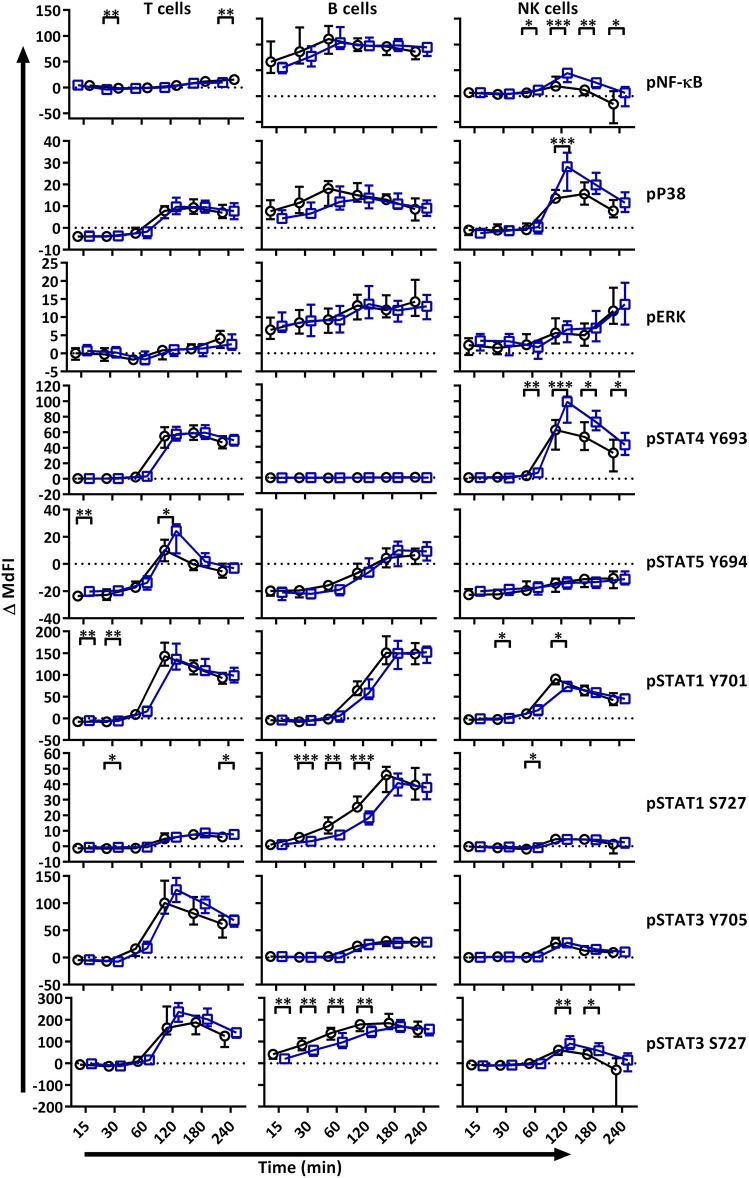
TLR stimulation results in different phosphorylation profiles in B cells, T cells, and NK cells of pSS patients compared to healthy controls. Phosphorylation levels of NF-κB, P38, ERK1/2, STAT4 Y693, STAT5 Y694, STAT1 Y701, STAT1 S727, STAT3 Y705, and STAT3 S727 were analyzed by flow cytometry at different time points after stimulation with TLR7 and −9 ligands. Comparisons of change of phosphorylation levels (ΔMdFI) between pSS patient (black) and healthy donors (blue) are given. Comparisons between pairs were done using an Unpaired Mann-Whitney test. Line graphs show the median and 25–75 percentiles. Differences were considered statistically significant when *p* ≤ 0.05, with significance indicated as * ≤ 0.05, ** ≤ 0.01, *** ≤ 0.001 and **** ≤ 0.0001. The data represents 25 healthy controls and 25 patients pooled from 13 independent experiments.

We next analyzed MAPK/ERK and JAK/STAT signaling networks upon TLR7 and −9 stimulation of PBMC. Initial responses (15–60 min) were weak relative to respective basal measurements in both T and NK cells, with little or no change observed in phosphorylation of the measured epitopes (Figure 2). The strongest initial responses were seen in B cells for NF-κB, P38, STAT1 S727, and STAT3 S727. The induction of phosphorylation of STAT1 S727 and STAT3 S727 in B cells from pSS patients was significantly stronger than healthy donor cells. In order to exclude effects of the medication on the analyses, we removed medicated patients from the analyses. This resulted in an even more pronounced difference between pSS patients and healthy donors (Supplementary Figure S2).

After 60 min of TLR7 and −9 stimulation, many epitopes of pSS patients displayed altered phosphorylation pattern compared to healthy donors, independent of medication (Figure 2, Figure S2).

**Figure 3:**
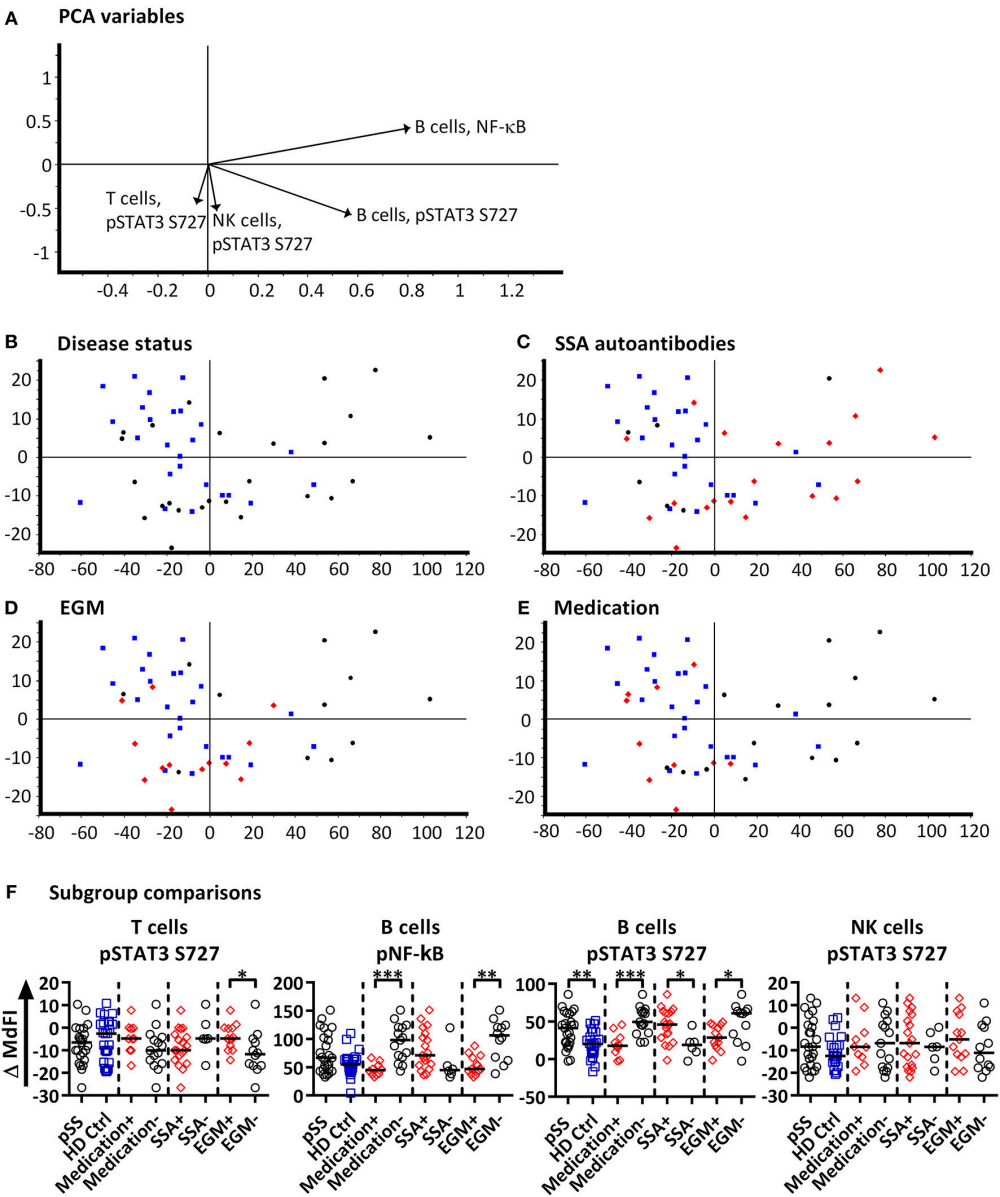
PCA analysis of induced phosphorylation in PBMC at 15 min following stimulation with TLR 7 and −9 ligands. Groupings of samples by PCA are shown by disease status **(B)**, SSA autoantibody positivity **(C)**, EGM presence **(D)**, and medication use (DMARDs or corticosteroids) **(E)**. Healthy donor samples are indicated by blue squares, pSS patients as black circles, and pSS patients with SSA autoantibodies, EGM or using prescribed DMARDs or corticosteroids (**C–E**, respectively) displayed as red diamonds. The loading plot, which contains information about the variable for the corresponding PCA is shown in **(A)**, with variables contributing to the PCA given as vectors. Variables contributing little to the PCA are plotted around the center as denoted by the gray axis, while variables that have high contributions are plotted further from the axes. After initial calculation of principal components the model was recalculated with only variable explaining >50% of the variance retained, stepwise reduction of less significant variables with low variable leverage was then performed. Scatter box plots of variable used in PCA for TLR7 and −9 ligand induced responses are given in **(F)**. Figures show change in MdFI from 0 to 15 min (Y axis) following addition of TLR7 and −9 ligands to PBMC cultures. Measured phospho-protein and responding cell type are labeled above each figure. Groups are identified at the base of each figure (X axis), initially with pSS patients (pSS) and healthy donors (HD Ctrl), pSS patients are further divided into SSA autoantibody positive and negative patients, patients with EGM (EGM+) or without EGM (EGM–), and unmedicated and medicated patients. Statistical comparisons were made between each of these pairs as indicated by dashed lines, with black bars representing medians. The data represents 25 healthy controls and 25 patients pooled from 13 independent experiments. Comparison between pairs were conducted using an Unpaired Mann-Whitney test with significance indicated as * ≤ 0.05, ** ≤ 0.01 and *** ≤ 0.001.

Next, we included phosphorylation profiles of TLR7 and −9 stimulated T, NK and B cells in the PCA. Phosphorylation levels after 15 min showed the strongest clustering of subgroups, while extended time course (>15 min) gave no additional resolution (Supplementary Figure S3), hence we focused on induced MdFI at 15 min (MdFI^15min^-MdFI^basal^) after stimulation with TLR7 and 9 ligands (Figure 3). PCA visualization showed a positive shift along PC1 for approximately half the pSS samples away from healthy donor samples (Figure 3B). The pSS samples that were distributed away from the healthy donors were largely composed of EGM-negative (Figure 3D) and unmedicated patients (Figure 3E). PC1 explained 83% of the variation with positive movement along PC1, strongly influenced by phosphorylation of NF-κB and STAT3 S727 in B cells (Figure 3A). PC2 explained 9% of the variation and was influenced primarily by induced phosphorylation of NF-κB in B cells in a positive direction and negatively by STAT3 S727 in NK, T and B cells (Figure 3A).

Further comparisons of variables used in the final PCA were conducted by Mann-Whitney U tests (Figure 3F). Comparisons between groups and subgroups (pSS patients, healthy donors, EGM+/–, SSA+/–) were analyzed with and without exclusion of medicated patients, in order to exclude that the effects seen were merely due to medication used by patients. EGM– patients had a significantly increased response to stimulation by TLR7 and −9 ligands in B cells through NF-κB compared to EGM+ patients. T cells from EGM-negative patients exhibited a significantly decreased response in STAT3 S727 compared to those from EGM+ patients. B cells showed a significantly increased response in STAT3 S727 in pSS patients compared to healthy controls, SSA+ compared to SSA– patients and EGM– patients compared to EGM+ patients. Upon removal of medicated patients, in particular the B cell phospho-epitopes for NF-κB, pP38, and STAT3 S727 resulted in stronger and significant differences between healthy donors and pSS patients (Supplementary Figure S4).

To summarize, after omitting patients prescribed DMARDs or corticosteroids from the analysis, B cells from pSS patients showed an increased response to TLR7 and −9 stimulation through NF-κB.

### Phosphorylation Profiles of Immune Cells Allow for Stratification of Patient Subgroups

**Figure 4:**
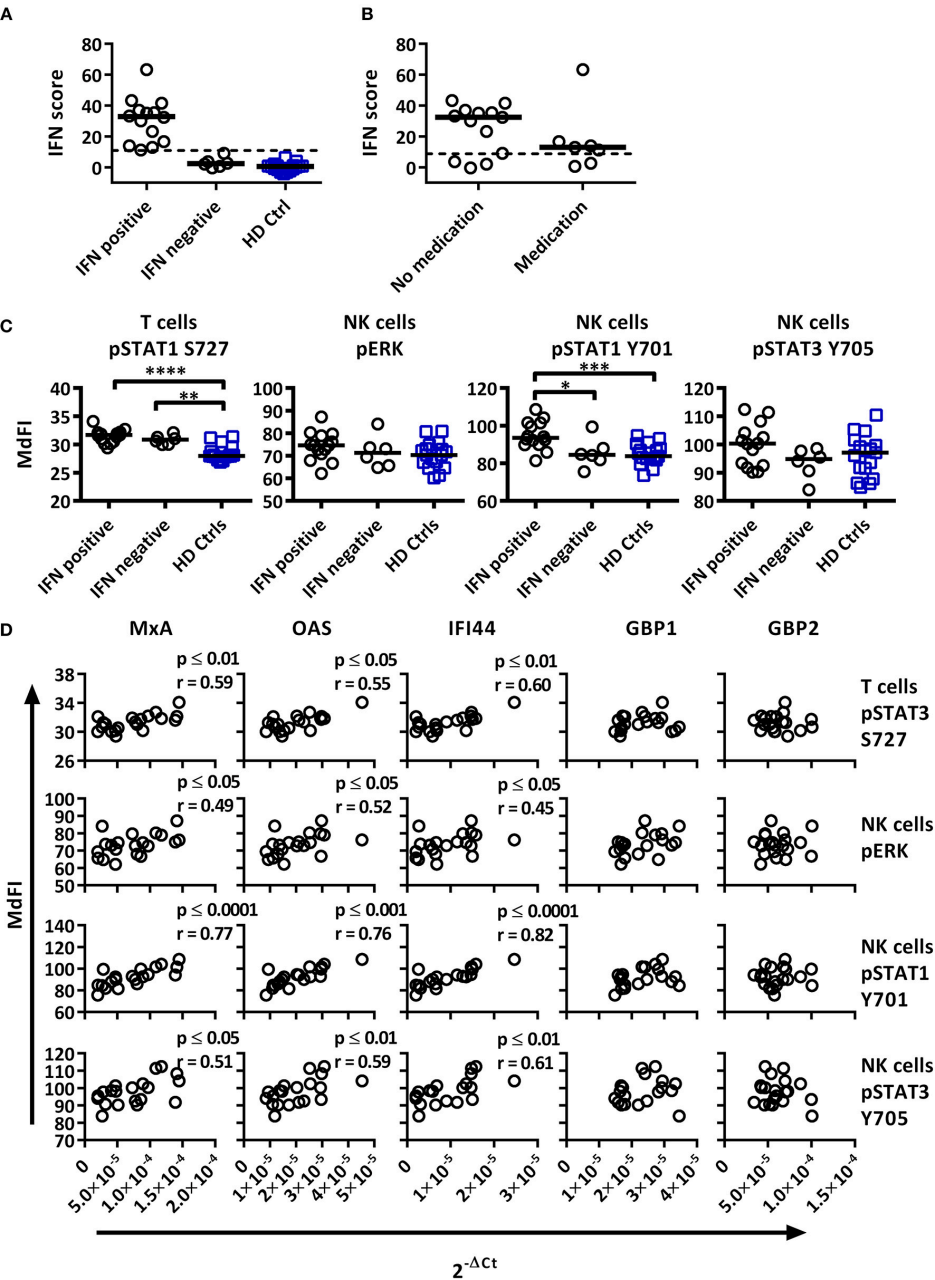
Associations of the basal phosphorylation profile with type I IFN inducible gene expression in pSS patients and healthy controls. IFN score was calculated based on standardized expression levels of three type I IFN inducible genes (MxA, OAS1, IFI44). A threshold was set to 8.8 based on 3 × SD of healthy controls as seen in **(A)**. Association between IFN score and the use of DMARD or corticosteroids is seen in **(B)**. Unpaired Mann-Whitney test comparisons of basal phosphorylation levels for IFN+ patients (*n* = 14), IFN– patients (*n* = 6), and healthy controls (*n* = 17) with the strongest associations are given in **(C)**, with medians indicated by black bars. Comparisons were conducted using an Unpaired Mann-Whitney test. Correlations between three type I IFN inducible genes (*MxA, OAS1, IFI44*) and two type II inducible genes (*GBP1, GBP2*) with basal phosphorylation levels in pSS patients (*n* = 20) epitopes, as given in **(C)**, is shown in **(D)**. Correlations were assessed with Spearman's rank test, with outliers removed using robust regression and outlier removal (ROUT) method and a ROUT coefficient Q of 1 was used. Significant values are indicated as * ≤ 0.05, ** ≤ 0.01, *** ≤ 0.001, and **** ≤ 0.0001. The flow cytometric data represents 17 healthy controls and 20 patients pooled from 13 independent experiments, with real time qPCR data representing a single experiment incorporating the 17 healthy controls and 20 patients.

A subgroup of pSS patients is characterized by a so-called type I IFN signature that correlates with increased disease activity (9). We therefore calculated an IFN score using three type I IFN inducible genes (*MxA, OAS, IFI44*) according to Feng et al. (19). As control, two type II IFN inducible genes (*GBP1, GBP2*) were included. The threshold was set to 8.8 based on 3 × SD of healthy controls. A type I interferon signature was found in 70% of patients and 0% of controls (Figure 4A). Medicated patients tended toward reduced expression compared to unmedicated patients (Figure 4B).

**Figure 5:**
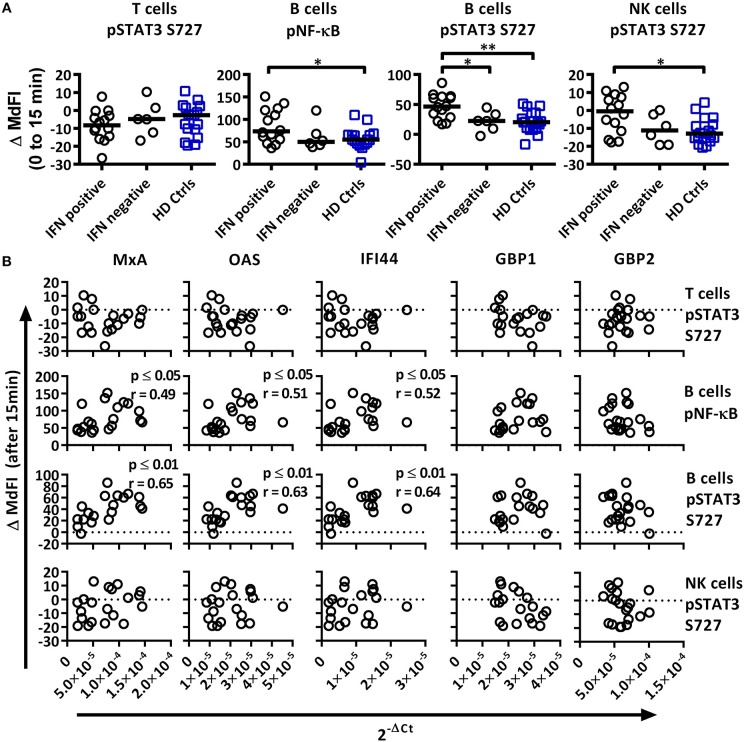
Associations of the phosphorylation profile 15 min after TLR7 and −9 stimulation with IFN inducible gene expression in pSS patients and healthy controls. **(A)** Unpaired Mann-Whitney test comparisons of phosphorylation levels after TLR7 and −9 stimulation for IFN signature positive patients (*n* = 14), IFN signature negative patients (*n* = 6) and healthy controls (*n* = 17), medians are given by black bars. **(B)** Correlation plots of variable identified by PCA and three type I IFN inducible genes (*MxA, OAS1, IFI44*) and two type II IFN inducible gene (*GBP1, GBP2*) for pSS patients (*n* = 20). Correlations were assessed with Spearman's rank test, with outliers removed using robust regression and outlier removal (ROUT) method and a ROUT coefficient Q of 1 was used. Significant values are indicated as * ≤ 0.05 and ** ≤ 0.01. The flow cytometric data represents 17 healthy controls and 20 patients pooled from 13 independent experiments, with real time qPCR data representing a single experiment incorporating the 17 healthy controls and 20 patients.

To investigate whether type I IFN activation was reflective of phosphorylation levels of intracellular signaling proteins, the gene expression was correlated to signaling profiles (Figures 4, 5). Comparisons of patients subdivided into type I IFN signature positive (IFN+) and negative (IFN–) patients and healthy donors were made for each intracellular signaling molecule and cell type, with basal phosphorylation variables that showed the strongest associations with type I IFN gene expression shown in Figure 4C. Both, IFN+ and IFN– patients displayed increased phosphorylation of STAT1 S727 in T cells. Interestingly, an increased phosphorylation of STAT1 Y701 was detected in NK cells of IFN+ patients compared to IFN–patients.

Basal phosphorylation levels in B cells from pSS patients were generally negatively correlated with type I IFN inducible gene expression, while positively correlated in NK and T cells, with weaker associations found with type II IFN inducible gene expression. The strongest significant correlations between type I IFN regulated genes were observed in NK cells for pERK, pSTAT1 Y701 and pSTAT3 Y705, and T cells for pSTAT1 S727 (Figure 4D). No significant associations were observed against type II IFN regulated genes for the aforementioned epitopes (Figure 4D).

We next analyzed the phosphorylation status upon TLR7 and −9 stimulation in correlation to IFN inducible gene expression in pSS patients against variables identified previously by PCA (Figure 5A). IFN+ patients showed increased phosphorylation of NF-κB and STAT3 S727 in B cells, and these differences remained significant following the removal of medicated patients. No difference was seen between the patient groups for phosphorylation of STAT3 S727 in T or NK cells.

Especially in B cells a prevalent positive correlation of phosphorylation levels and IFN inducible gene expression were detected. The strongest correlations were seen for phosphorylation levels of NF-κB and STAT3 S727 (Figure 5B). STAT4 Y693, NF-κB, P38, and STAT3 Y705 in NK cells and NF-κB in T cells showed positive associations, but only NF-κB and P38 reached statistical significant correlations. Of the other two variables identified by PCA, STAT3 S727 in T and NK cells, no significant correlation with gene expression was observed (Figure 5B).

### Plasma Cytokine Levels Correlate With Presence of Autoantibodies and Signaling Responses in Patient Subgroups

The role of cytokines in pSS has been a matter of great interest over the past few years (25, 26). Our aim was to examine the plasma cytokine concentration of our pSS patient cohort, compare them to healthy controls, and possibly correlate them to clinical parameters and phosphorylation pattern of the epitopes included in this study.

GM-CSF, IL-5, and IL-8 were excluded from the analyses as they were below detection limit in most samples analyzed. Eotaxin, IL-7, IP10, and MIG were not included in the analysis as they were not recommended to be measured in heparin plasma by the manufacturer.

**Figure 6:**
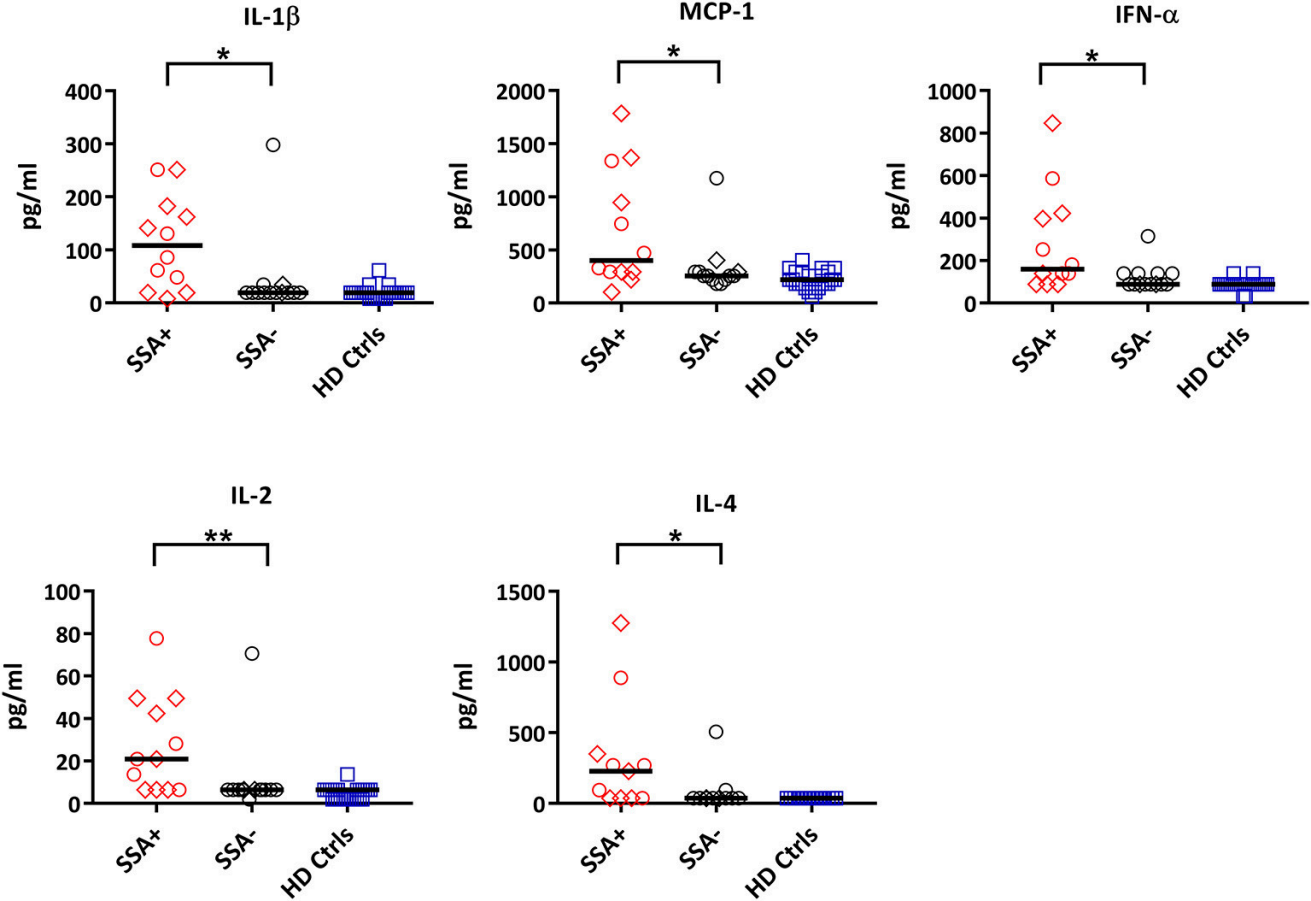
Differential expression of cytokines between autoantibody positive (SSA+) and autoantibody negative (SSA-) pSS patients. Cytokine profiles were measured by 25-plex Luminex assay in plasma. Cytokine levels (pg/ml) showing significant differences between SSA+ patients (red) and SSA– patients (black) are shown. Healthy controls (HD Ctrls) are shown in blue. Medicated patients are shown as diamond. Comparison between pairs was done by unpaired Mann-Whitney test between the two patient subgroups. Differences were considered statistically significant for *p* ≤ 0.05, with significance being indicated as * ≤ 0.05, ** ≤ 0.01. The median is indicated. The data represents pSS patients (*n* = 25) grouped into SSA+ patients (*n* = 12) and SSA– patients (*n* = 13) (except for IL-4, *n* = 11 in each category).

When comparing patients with controls, 12 out of the 25 cytokines measured were significantly upregulated in patients including IL-1ß, IL-13, IL-6, IL-12, MIP-1α, MIP-1ß, MCP-1, IL-15, IFN-α, TNFα, IL-2, and IL-4 (Supplementary Figure S5, Supplementary Table S5). When dividing the patients into subgroups based on the presence or absence of autoantibodies (SSA+/SSA–), extraglandular manifestations (EGM+/EGM–), IFN score, medication and Focus score, the only significant differences were seen in SSA+ patients, where IL-1ß, MCP-1, IFN-α, IL-2, and IL-4 were significantly upregulated compared to SSA– patients (Figure 6, Supplementary Table S6).

**Figure 7:**
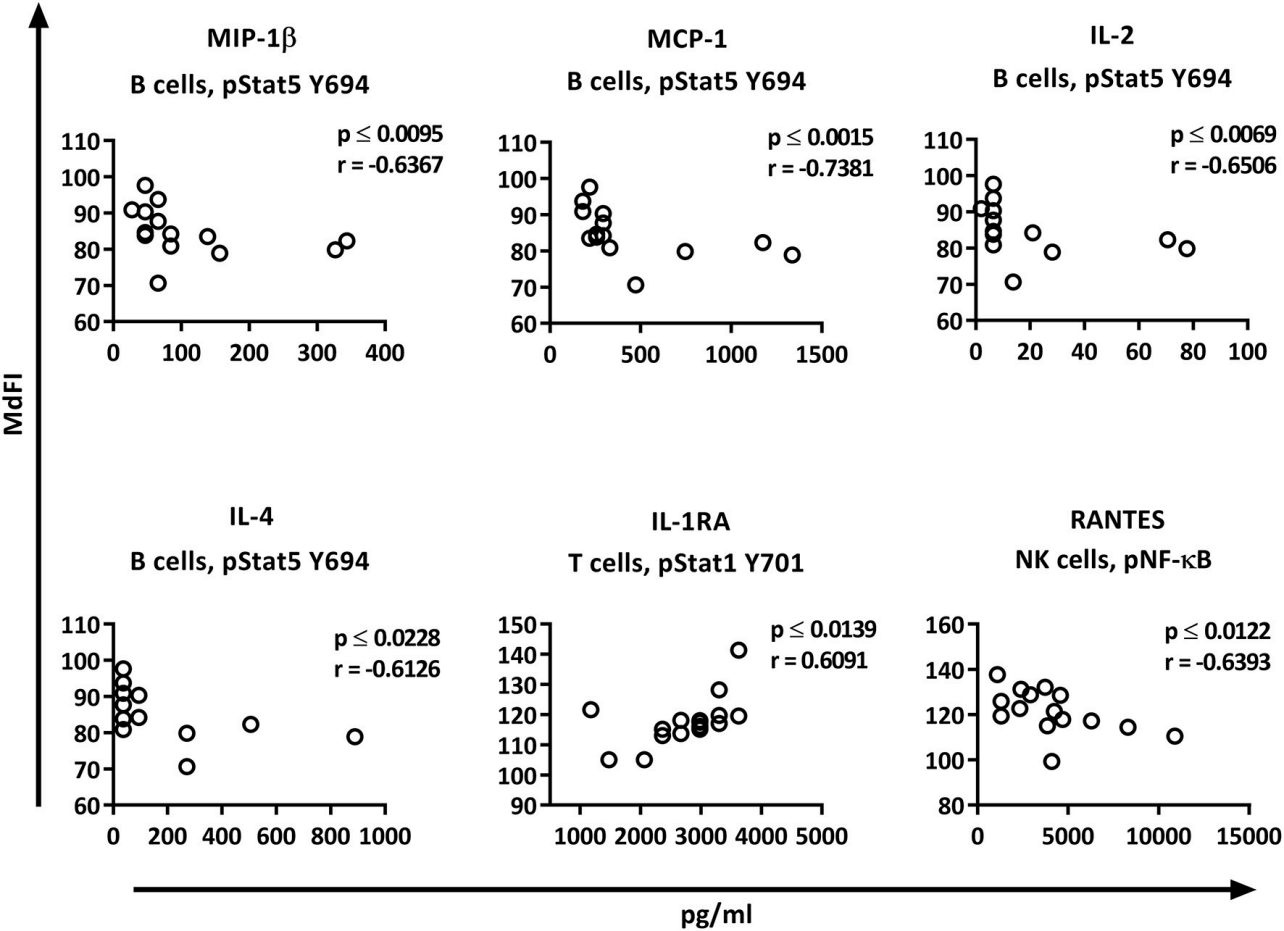
Correlations of basal phosphorylation profiles with plasma cytokine levels in pSS patients without medication. Correlations were assessed by Spearman's rank test. Strong (*r* = 0.6–0.79) to very strong (*r* = 0.8–1.0) associations are shown with respective *p* and *r*-values given at the side of each graph. Correlations were considered statistically significant for *p* ≤ 0.05. X-axis denotes concentration of cytokines (pg/ml) and Y-axis denotes phosphorylation levels (MdFI). The data represents pSS patients (*n* = 16) without medication (except for IL-4, *n* = 14, and RANTES, *n* = 15).

Correlation analysis of the individual cytokines of the patients to the phosphoproteins yielded significant results. While the basal phosphorylation profiles of all the patients showed only moderate correlations (< 0.6) to the plasma cytokine concentrations, excluding medicated patients from the analysis resulted in strong to very strong correlations of RANTES to pNF-κB in NK cells, MIP-1ß, MCP-1, IL-2, and IL-4 to pSTAT5 Y694 in B cells, and IL-1RA to pSTAT1 Y701 in T cells (Figure 7).

**Figure 8:**
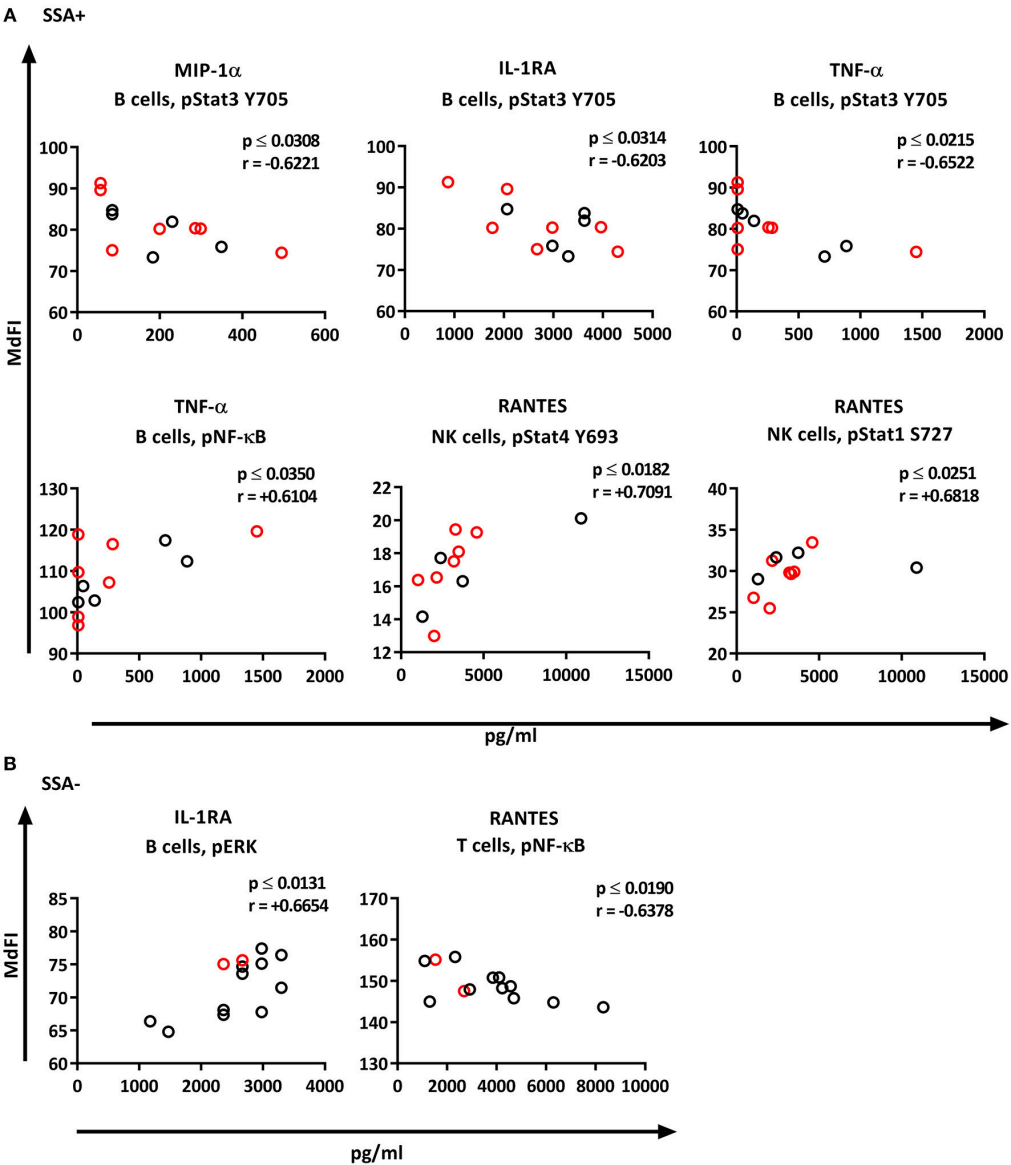
Correlation of basal phosphorylation status with plasma cytokine levels in pSS patients grouped according to autoantibody status. Autoantibody positive pSS patients (SSA+) **(A)**, autoantibody negative pSS patients (SSA–) **(B)**. Correlations were assessed by Spearman's rank test. Medicated patients are shown in red while un-medicated patients are shown in black. Strong (*r* = 0.6–0.79) to very strong (*r* = 0.8–1.0) associations are shown with respective *p* and *r*-values given at the side of each graph. Associations were considered statistically significant for *p* ≤ 0.05. X-axis denotes concentration of cytokines (pg/ml) and Y-axis denotes phosphorylation levels (MdFI). The data represents pSS patients (*n* = 25) grouped into SSA+ patients (*n* = 12) (except for RANTES, *n* = 11) and SSA– patients (*n* = 13).

**Figure 9:**
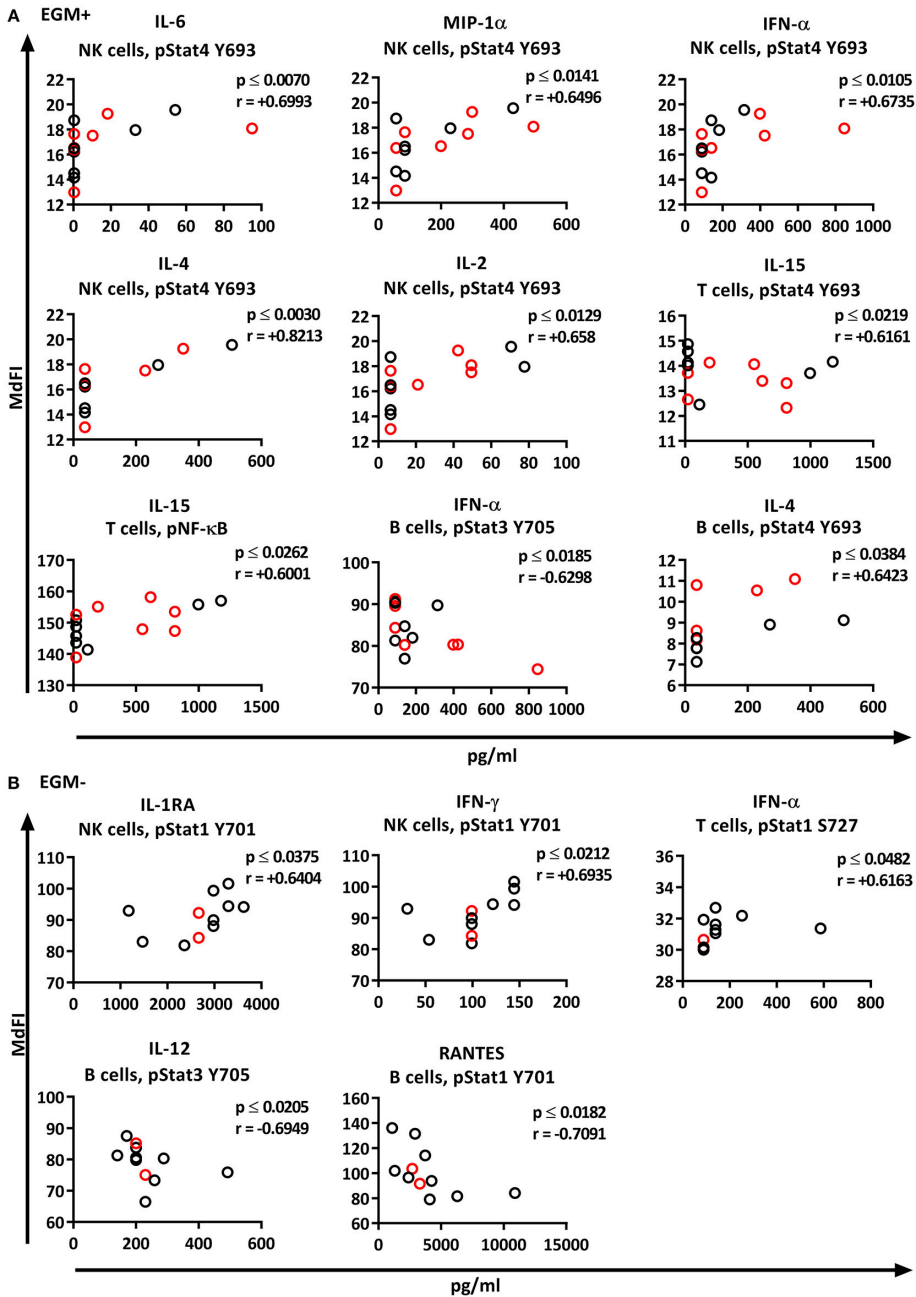
Correlation of basal phosphorylation profiles with plasma cytokine levels in pSS patients grouped according to the presence of extraglandular manifestations (EGM). Extraglandular manifestation positive pSS patients (EGM+) **(A)** and extraglandular manifestation negative pSS patients (EGM–) **(B)**. Correlations were assessed by Spearman's rank test. Medicated patients are shown in red while un-medicated patients are shown in black. Strong (*r* = 0.6–0.79) to very strong (*r* = 0.8–1.0) associations are shown with respective *p* and *r*-values given at the side of each graph. Associations were considered statistically significant for *p* ≤ 0.05. X-axis denotes concentration of cytokines (pg/ml) and Y-axis denotes phosphorylation levels (MdFI). The data represents pSS patients (*n* = 25) grouped into EGM+ patients (*n* = 14) (except for IL-4, *n* = 11) and EGM– patients (*n* = 11).

We next explored the correlation of the plasma cytokine levels to basal phosphorylation pattern depending on presence or absence of SSA and EGM. The exclusion of medicated patients in the subgroup analysis resulted in too few patients per group for reliable data, therefore all patients were included in this part of the analysis. We observed strong to very strong correlations in SSA+ patients of MIP-1α, IL-1RA, and TNF-α to pSTAT3 Y705 in B cells, TNF-α to pNF-κB in B cells, and RANTES to pSTAT4 Y693 and pSTAT1 S727 in NK cells (Figure 8A). In SSA– patients, RANTES correlated to pNF-κB in T cells and pERK in T cells (Figure 8B). Patients with EGM had strong to very strong correlations of several cytokines to amongst other pSTAT4 Y693 in NK (Figure 9A), and RANTES, IFN-γ, IL-1RA, IFN-α, and IL-12 correlated with various phospho-epitopes in EGM– patients (Figure 9B).

## Discussion

Autoimmune diseases often exhibit skewed cytokine and gene expression profiles. Elucidating mechanisms that contribute to these profiles are crucial in understanding the pathogenesis of autoimmune disease. Of prominent interest in autoimmunity is an increased expression of type I IFN regulated genes known as the “interferon signature” which has been observed in various autoimmune diseases (8, 9, 27, 28). Continuous activation and dysregulation of TLR and type I IFN signaling have been speculated to play a part in this signature and pathogenesis of autoimmune disease (29), and IFN signature positive pSS patients have been shown to have increased expression of TLR7 in certain cell types (30). In addition, we have previously shown that PBMC of pSS patients have an altered response to IFN-α stimulation (10). Hence we investigated cell signaling profiles in PBMC of pSS patients upon stimulation via TLR7 and −9, determined the gene expression profile of several IFN inducible genes and correlated these findings to plasma cytokine levels.

In accordance with a previous observation we found increased basal STAT5 Y694 phosphorylation in T cells from pSS patients compared to healthy donors (31). However, in contrast to our study, the authors also found significant differences in B cells for basal phosphorylation of STAT5 Y694 and no differences in phosphorylation of STAT1 Y701 in T cells (31). Another study also reported on significant differences in basal phosphorylation levels of STAT3 Y705 in T cells (32), which also is in contrast to our findings. However, these differences are likely the result of the use of cryopreserved PBMC and long culture period (6 h) in our study, as both other studies used freshly isolated cells.

Although PCA using basal measurements allowed for grouping of pSS patients and healthy donors, its use to identify important variables of basal measurements for subgrouping of the patient cohort was largely unsuccessful. The majority of B cell associated variables largely correlated with each other, as did T and NK cells with the grouping of pSS patients being a consequence of higher basal phosphorylation in T and NK cells. Even though some differences were seen when dividing the pSS patients according to medication, the small sample size limits how much we can speculate on the pathophysiological significance of this.

Basal STAT1 Y701 in NK cells was increased in type I IFN+ patients, but the difference was no longer significant when excluding medicated patients. However, the sample size following exclusion was relatively low, while the data spread remained similar. Further, phosphorylation of many of the measured phospho epitopes in NK and T cells from pSS patients, in particular STAT1 Y701 in NK cells, were positively correlated with the expression levels of the three type I IFN inducible genes, while in B cells a negative correlation was observed. In contrast, little relationship was observed for the type II IFN induced genes. Not much is known about NK cells in Sjögren's syndrome, so the correlation with basal phosphorylation of ERK, STAT1 Y701, and STAT3 Y705 in NK cells is especially interesting. Moreover, levels of several plasma cytokines also correlated with basal phosphorylation in NK cells. Further studies are required to confirm these correlations.

Following stimulation with TLR7 and −9 ligands, B cells from pSS patients showed a significantly increased STAT3 S727 response compared to healthy donors. After excluding medicated patients, phosphorylation of NF-κB and P38 was also significantly elevated in B cells from pSS patients compared to healthy donors. These findings support the notion that pSS patients display a hyperactive B cell response and are in line with our previous study showing increased expression of IFN-α in B cells from pSS patients after incubation with TLR7 ligands compared to B cells from healthy donors (33). The increased response to TLR7 and −9 ligands through these pathways may play a role in the increased expression of IFN-α from B cells of pSS patients, and may also contribute to the observed IFN signature in some pSS patients. Thereby, it opens for speculations regarding the importance of viral infections for pSS patients. Further, a number of polymorphisms associated with pSS and the presence of autoantibodies in pSS could potentially affect signaling through NF-κB, P38, and STAT3 S727. If the potentiation of these signaling profiles are associated with polymorphisms in negative regulators of TLR signaling, including A20 (antiapoptotic signaling protein) which deubiquitylates TRAF6 (tumor-necrosis factor-receptor-associated factor 6), and affects both MyD88-dependent and MyD88-independent pathways (34), these differences will also likely be reflected in other cell types using the same pathways. Alternatively, the increased response through these pathways may be attributed to the cellular effects of induction of type I IFN gene expression.

Interestingly, 70% of the patients included in this study had an activated type I IFN system. This is somewhat higher than previously reported for pSS patients [around 55%; (9)] and SLE patients [around 50%; (27)]. This might be due to limited sample size and differences in patient inclusion criteria. However, also the plasma levels of IFN-α were elevated in our cohort of pSS patients, especially in SSA+ patients, which might explain the high percentage of IFN+ patients.

Induced phosphorylation of STAT3 S727, NF-κB, and P38 correlated significantly with type I IFN inducible gene expression. Type I IFN has been shown to enhance B cell responses to TLR7 ligands and upregulate TLR7 and MyD88 expression in naïve B cells (35, 36). Increased type I IFN gene expression may therefore act to potentiate these signals.

Systemic autoimmune diseases are associated with the production of autoantibodies and have an important role in the immunopathogenesis of various autoimmune diseases (29). Animal models have indicated links between TLR recognizing nucleic acids and the production of nucleic acid recognizing antibodies (37). Additionally, type I IFN inducible gene expression has been observed to positively correlate with titers of SSA and SSB autoantibodies in SS (38). We showed increased responses of B cells from SSA+ pSS patients through phosphorylation of STAT3 S727 in response to TLR7 and −9 stimulation compared to SSA– patients. Our study thereby links all three observations, enhanced TLR7 and −9 responses, increased type I IFN gene expression and autoantibodies, further highlighting their importance in autoimmunity.

Principal component analysis suggests that it is possible to subdivide pSS patients based on presence of EGM. EGM negative patients displayed enhanced TLR responses through NF-κB, P38, and STAT3 S727 in B cells compared to EGM+ patients. This was also seen after removal of patients prescribed the glucocorticoid prednisone, which has been reported to inhibit NF-κB activation (39), and hydroxychloroquine (Plaquenil®) inhibiting TLR7 and −9 signaling (40). However, it is still surprising that it was the EGM negative patients that had an enhanced response in B cells, as a number of EGM in SS are associated with high prevalence of hyperreactive B-cells as well as SSA and SSB autoantibodies (41). One possible explanation might be that the lower responses of B cells from EGM+ patients represent movement of more reactive B cells from the periphery to other compartments not being analyzed in this study.

Several plasma cytokines correlated significantly with basal phosphorylation levels of various phospho-epitopes in T-, B-, and NK cells. However, even though presence of outliers was tested using ROUT's method, most outliers detected by the test were not excluded from the analyses except a few very obvious ones, as Rout's method is not very reliable for non-parametric data. The low number of patients per subgroup further requires caution concerning interpretation of the data. A larger number of patients has to be analyzed before a more reliable correlation between phosphorylation pattern, cytokine profile, presence of autoantibodies and EGM might be found. This might also help clarifying the pathophysiological relevance of our findings.

This study has a number of limitations, for one, small sample size, which is further affected by the heterogeneity of the patients, and in particular the number of medicated patients. Second, as this was a pilot study, the analysis was limited to the three main subsets of lymphocytes (T, B, and NK cells). As these cell subsets are made up of numerous other subtypes, differential responses and shifts in their relative frequency in the peripheral blood may affect cellular responses. Immunophenotyping studies have shown altered distribution of various cell types in peripheral blood (42, 43). We can therefore not be certain that the changed signaling profiles are not caused by these alterations rather than potentiated or repressed signaling. Moreover, certain subpopulations might be more prone to apoptosis upon longer stimulation with TLR7 and −9 ligands, which we did not address in this study. Finally the type I and II IFN regulated gene expression was assessed in PBMC, and assessment for each cell type might have strengthened associations and be more informative in determining origin of the signature. In addition, some of the statistically significant differences were rather small. Future studies will have to address the biological relevance in more functional assays.

In conclusion, we have identified increased responses by B cell from pSS patients to TLR7 and −9 stimulation through STAT3 S727 and NF-κB. The increased response was found to correlate to a type I IFN signature. The results suggest that the type I IFN signature may either induce or in part be derived in response to increased activation of NF-κB and STAT3 S727 upon TLR7 or −9 activation, facilitating increased production of interferon.

## Author Contributions

PV, RJ, and SA conceived of study. RD, PV, and SA designed the study. RD and BB processed PBMC samples and conducted flow cytometric analysis. SA conducted real-time quantitative PCR. IS and SMS performed cytokine assays. RD, BB, SG, IS, and SA analyzed and processed the data. DH and JGB selected patients and collected patient data. RD and SA drafted the manuscript. All authors revised the manuscript and approved the final version.

### Conflict of Interest Statement

The authors declare that the research was conducted in the absence of any commercial or financial relationships that could be construed as a potential conflict of interest.
