# Supplementary material for: Single Cell Based Phosphorylation Profiling Identifies Alterations in Toll-Like Receptor 7 and 9 Signaling in Patients With Primary Sjögren's Syndrome
Source: Front Immunol. 2019 Feb 21;10:281. doi: 10.3389/fimmu.2019.00281 (PMC6393381; doi:10.3389/fimmu.2019.00281)
Supplement: Supplementary file 1 [file Presentation_1.pdf]

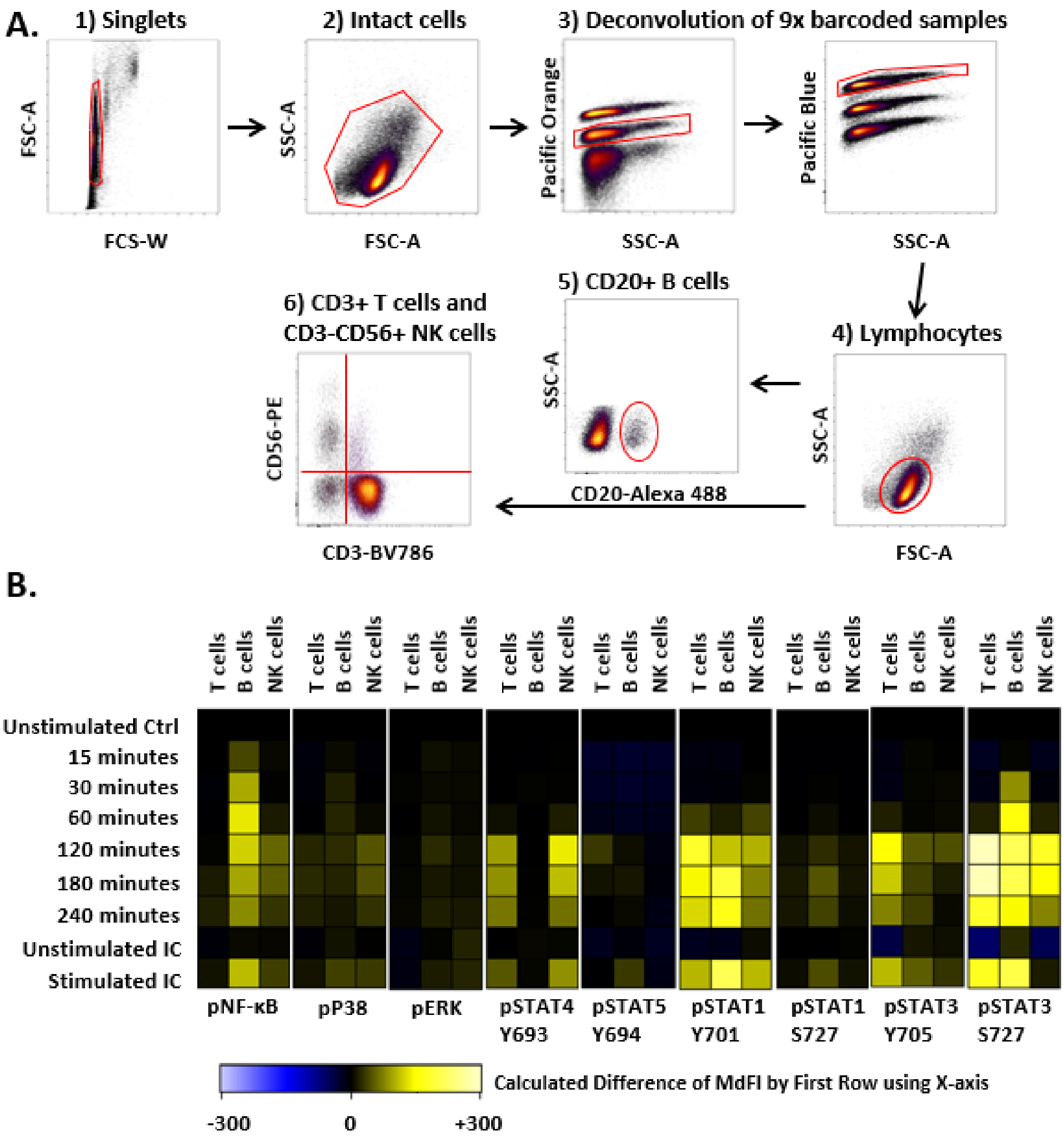

Figure S1. A. Representative gating strategy used in the analysis of intracellular signaling pathways in PBMC. Single cells were gated based on their forward scatter area (FSC-A) and forward scatter width (FSC-W), followed by intact cells based on side scatter area (SSC-A) and FSC-A. The different stimulation conditions were then identified through the intensities of their pacific orange and pacific blue stains. Lymphocytes were identified based on their FSC-A and SSC-A scatter properties. Lymphocytes were then subtyped as B cells (CD20+), T cells (CD3+CD56-) or NK cells (CD3-CD56+) based on surface antigen expression. **B. Cell type specific signalling profile for a single donor following stimulation with TLR7 and -9 ligands over a 4 hour time course**. The figure indicates the change of MdFI compare to an unstimulated sample for each phospho-epitope.

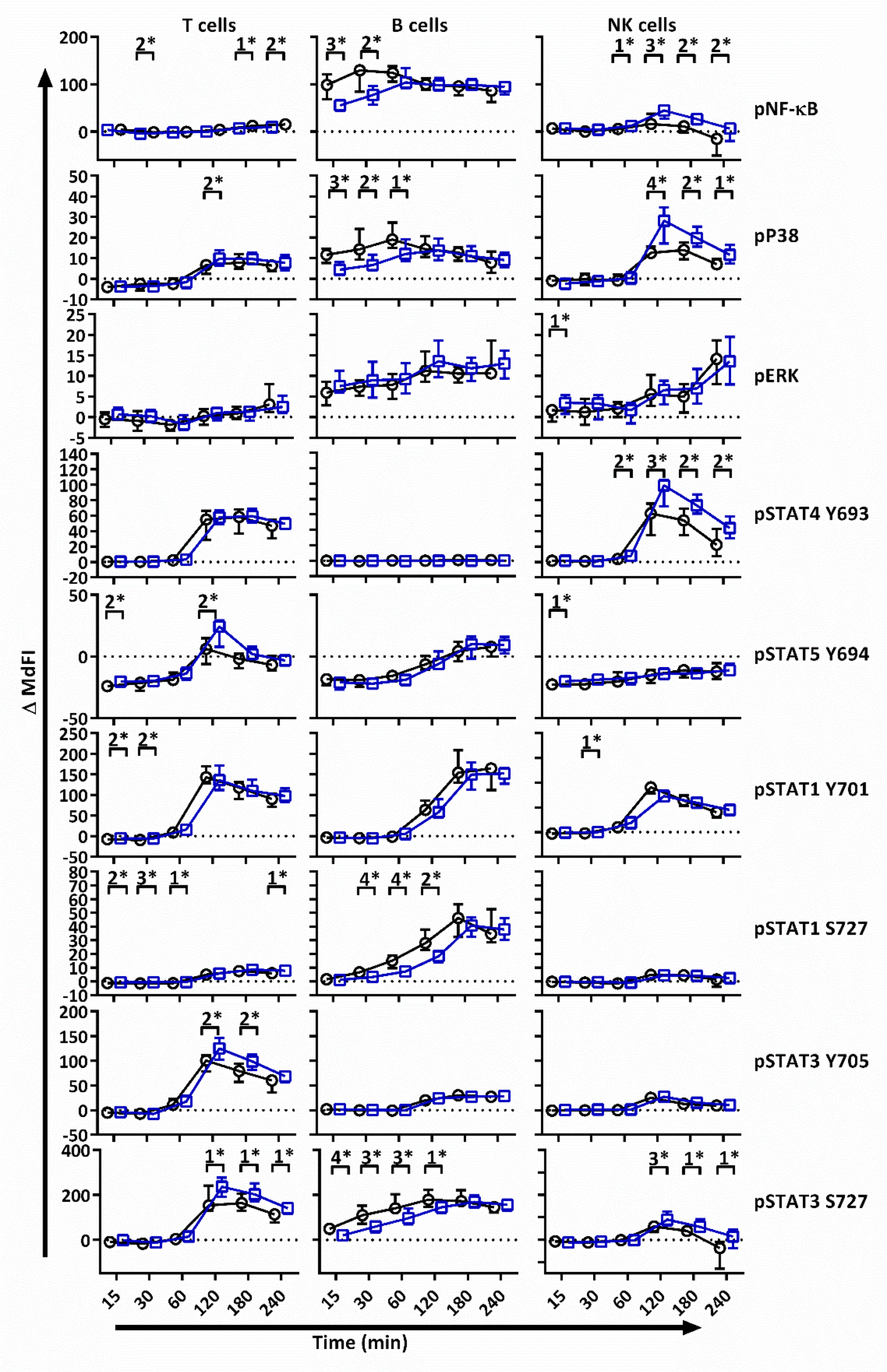

Figure S2. **Phosphorylation profiles in B cells, T cells and NK cells of unmedicated pSS patients compared to healthy controls** (see also Figure 2). Phosphorylation levels of NF-κB, P38, ERK1/2, STAT4 Y693, STAT5 Y694, STAT1 Y701, STAT1 S727, STAT3 Y705 and STAT3 S727 were analysed by flow cytometry at different time points after stimulation with TLR7 and -9 ligands. Comparisons of change of phosphorylation levels (ΔMdFI) between unmedicated pSS patient (black) and healthy donors (blue) are given. Comparisons between pairs were done using an Unpaired Mann-Whitney test. Line graphs show the median and 25 to 75 percentiles. Differences were considered statistically significant when $p \leq 0.05$, with significance indicated as 1* ≤ 0.05, 2* ≤ 0.01, 3* ≤ 0.001 and 4* ≤ 0.0001.

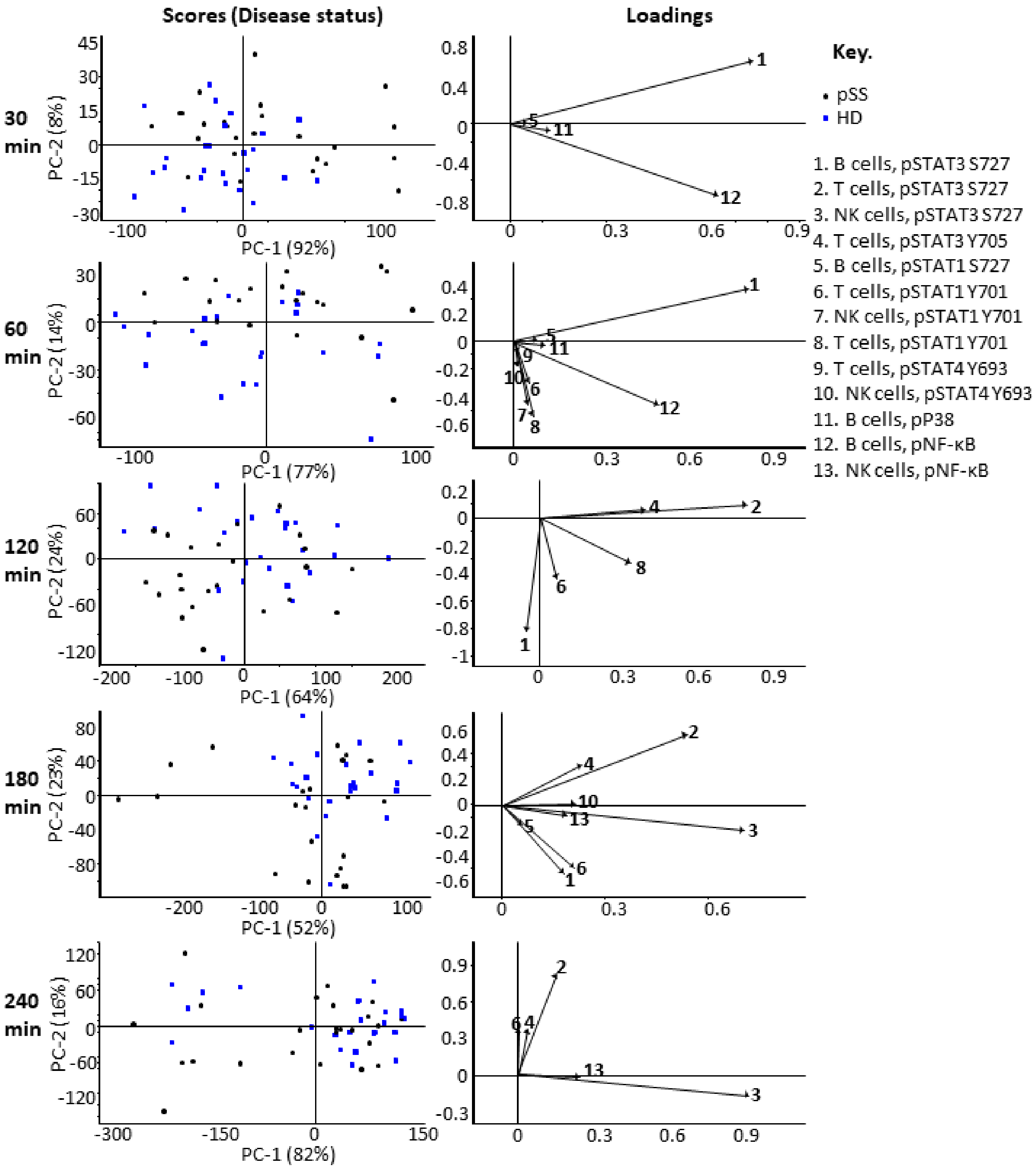

Figure S3. **Groupings of samples by PCA for time points 30, 60, 120, 180 and 240 min**. Black circle: pSS patient, blue square: healthy donor. The figure key is shown on the right side. The loading plot containing information about the variables for the corresponding PCA is shown on the right, with variables given as vectors. Variables contributing little to the PCA are plotted around the center as denoted by the axis, while variables having high contributions are plotted further from the axes.

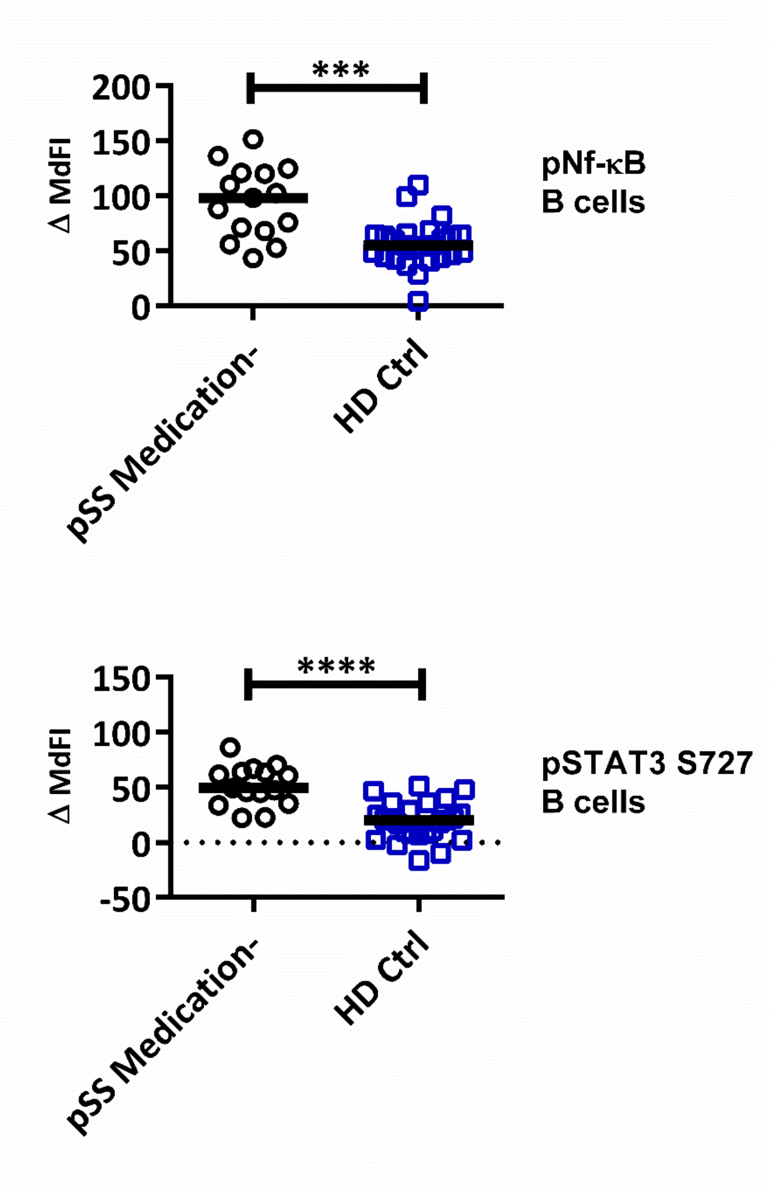

Figure S4. **Comparison of unmedicated patients to healthy controls as shown in Figure 3F**. Unpaired Mann Whitney tests between healthy donors and un-medicated patients give significant differences for both change of pNf-κB and STAT3 S727 in B cell after 15 minutes with p-values of 0.0002 (median: pSS- 98.22, HD 55.09; 95% CI: 16.04 to 59.98) and > 0.0001 (median: pSS- 49.39, HD 19.97; 95% CI: 20.15 to 44.88) respectively. Black circle: unmedicated patients; blue square: healthy controls.

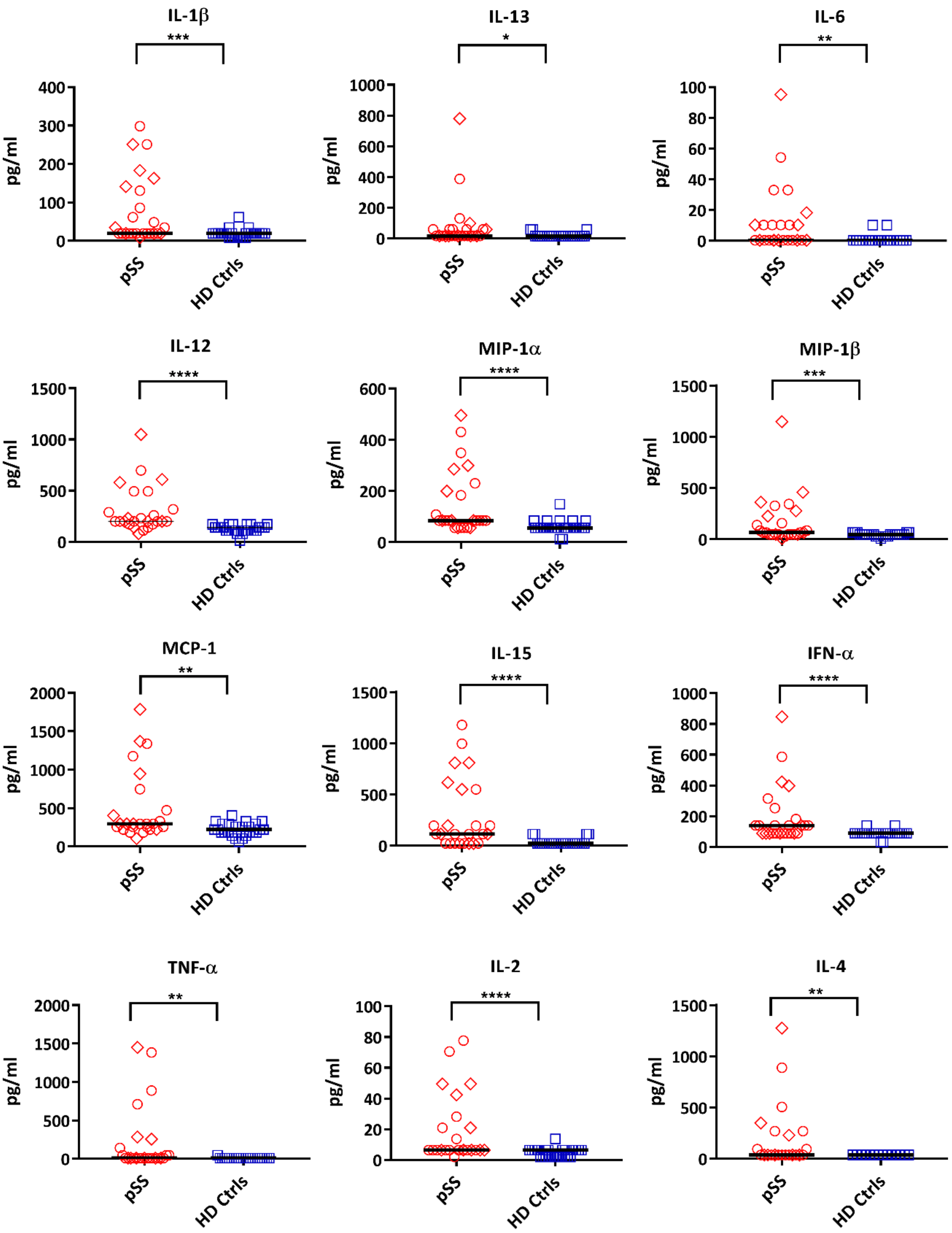

Figure S5. Variation in plasma cytokine concentrations between pSS patients and healthy controls. Cytokine levels (pg/ml) were measured by 25-plex Luminex assay in plasma and significant differences between patients (pSS; red) and healthy controls (HD Ctrls; blue) are shown. Medicated patients are shown as diamond. Comparison between pairs was done by unpaired Mann-Whitney test. Differences were considered statistically significant for p values ≤ 0.05, with significance indicated as * ≤ 0.05, ** ≤ 0.01, *** ≤ 0.001 and **** ≤ 0.0001. Medians are shown by line graphs. The data represents pSS patients (n=25) (except for IL-15 and IL-4 where n= 21 and 22 respectively) and HD Ctrls (n=25) (except for IL-6, TNF-α and IL-4 where n=24, 23 and 16 respectively).

Table S1. Relevant information for repeating the experiment as presented in "The minimum information about a Flow Cytometry Experiment (MIFlowCyt)".

Data set	Sample/Reagent/ Controls/Instrument	Details
**Samples/Specimens**	Patient samples	*Species: Homo Sapiens, Sex: female, Median age (range): 56 (33-73), Phenotype: pSS, Location: Department of Rheumatology, Haukeland University Hospital, Bergen, Norway. Collection methodology: Venule puncture*
	Healthy donor samples	*Species: Homo Sapiens, Sex: female, Median age (range): 54 (42-70), Phenotype: Unknown, Location: Blood bank, Haukeland University Hospital, Bergen, Norway. Collection methodology: Venule puncture*
	Single donor control	*Species: Homo Sapiens, Sex: Unknown, Age: Unknown, Phenotype: Unknown, location: Bergen, Norway. Collection methodology: Venule puncture*
**Sample treatment**	Blood collection	*Lithium-heparin tubes for patients and healthy donor samples, and a citrate phosphate dextrose adenine blood collection bag for the single donor control*
	PBMC isolation	*PBMC isolated within 1hr of blood collection by density gradient centrifugation with lymphoprep (Axis-Shield, Cat#07861)*
	Cryopreservation	*Immediately following isolation, PBMC samples were washed 2x with PBS (Lonza, Cat# 17-516F) and frozen at 5x10^6 cells/ml in 7.5% DMSO (Sigma-Aldrich, Cat#D2650), 50% X-vivo 20™ (Lonza, cat#BE04-448Q), 42.5% Profreeze™ CDM NAO media (Lonza, cat# 12-769E) and at a cooling rate of 1 Celsius/min(CoolCell LX, Biocison, Cat#BCS-405) at -70°C overnight before long term storage in -150°C freezer*
	Thawing	*Cells were thawed rapidly at 37°C in a water bath, 1ml of 37°C X-vivo 20™ was added dropwise to each vial prior to addition to 9ml X-vivo 20™ and washed (300g) prior to culturing*
	Culturing	*The cells were cultured at 37°C with 5% CO2 at 3 x10 cell/ml in a Megablock® 96 well plate (Starstedt, Cat#82.1972.002). The cells were rested for 2 hours prior to stimulation.*
	Stimulation	*50µl of X-vivo 20™ (at 240 minute time point) or X-vivo20™ with TLR7 (CL097; Invivogen, cat#tlrl-c97-5) and -9 ligands (CpG type B ODN 2006 and type C ODN 2395; Invivogen, cat#tlrl-2006-1 and cat#tlrl-2395-1 respectively) at 2 µg/ml each according to a reverse time course for 15, 30, 60, 120, 180, or 240 minutes*
	Fixation	*Following stimulation cells were immediately fixed at room temperature for 10 minutes with 37°C 16% paraformaldehyde (Electron Microscopy Sciences, cat#15710) for a final concentration of 1.5%.*
	Permeabilization	*After fixation PBMCs were washed at 1000g for 5 min 4°C with PBS, and resuspended in 50µl. 1ml ice cold methanol (Sigma Aldrich, cat#32213-2.5L-M) was added dropwise. Cells were then incubated on ice for 30min, and then kept overnight at -80°C.*
	Barcoding	*Prior to barcoding cells were washed 1x with PBS. Cells were then stained for 30 minutes at 4°C with 3 levels of pacific orange and pacific blue succinimidyl ester dyes (PB 100, 25 and 6.3 ng/ml; PO 250, 70 and 0 ng/ml; Life Technologies, cat#P30253 and #10163 respectively) diluted in PBS.*
	Antibody staining	*Barcoded cells were then washed 1x in PBS containing 1% BSA (Sigma-Aldrich, cat#A7906-500g) before being combined into a single sample. The sample was washed (PBS with 1% BSA) and incubated with 2µl Fc receptor block (Miltenyi Biotec, cat#130-059-901) per 1x10^6 cells for 10 minutes on ice. The sample was then divided into 3 and incubated for 30 minutes at room temperature stained with 3 different antibody panels.*
	Antibody panels and staining concentrations	*Phospho-specific monoclonal antibodies- Alexa Fluor® 647 conjugated anti-STAT4 (pY693, clone 38/p-STAT4, panel 1, cat#558137, dilution- 1:10), anti-STAT1 (pS727, clone K51-856, panel 2, cat#560190, dilution- 1:10) and anti-STAT3 (pS727, clone 49/p-STAT3, panel 3, cat#558099, dilution- 1:20); PerCP-Cy™5.5 conjugated anti-ERK1/2 (pT202/pY204, clone 20A, panel 1, cat#560115, dilution- 1:10), anti-STAT1 (pY701, clone 4a, panel 2, cat#560113, dilution- 1:10) and anti-STAT3 (pY705, clone 4/P-STAT3, panel 3, cat#560114, dilution- 1:6.5); and PE-Cy™7 conjugated anti-NF-κB p65 (pS529, clone K10-895.12.50, panel 1, cat#560335, dilution- 1:20), anti-p38 MAPK (pT180/pY182, clone 36/p38, panel 2, cat#560241, dilution- 1:10) and anti-STAT5 (pY694, clone 47 / STAT5(pY694), panel 3, cat#560117, dilution- 1:10) (all from BD Biosciences). Cell surface markers BV786 conjugated anti-CD3 (clone SK7, BD HorizonTM, cat#563799, dilution- 1:100), Alexa Fluor® 488 conjugated anti-CD20 (clone H1 (FB1), BD Biosciences, cat#558056, dilution- 1:20) and PE conjugated anti-CD56 (clone N901, Beckmann Coulter, CA, USA, cat#A07788, dilution- 1:50)*
	Acquisition	*Following staining samples were washed 2x in PBS with 1% BSA and resuspended in PBS containing 1% BSA and 2mM EDTA (Sigma-Aldrich, cat#E7889-100ml) and then immediately analyzed on the flow cytometer.*

**Controls**	Flow cytometer calibration	*Flow cytometer calibrated daily with BD cytometer setup and tracking beads (BD Biosciences, cat#655051). Standardized consistent fluorescence intensity target values across experiments were obtained with "Application Setting" on the BD FACSDiva software*
	Single donor control	*Cryopreserved PBMCs from a single donor were included in each assay (n = 13) as a positive control, for inter-assay normalization and assessing assay to assay variability.*
	Biological control	*For each donor an unstimulated PBMC sample was analyzed to assess donor specific changes in the phosphorylation of phospho-epitopes follow IFNα stimulation*
	Compensation	*Single stained compensation controls were analyzed for each assay to assess and control for spectral spillover. Unstained / single stained beads (BD Biosciences, cat#552843) were used for compensation for antibody-fluorchrome conjugates; for amine reactive dyes (Pacific blue and Pacific Orange), unstained and single stained cells were used.*
**Instrument**	Instrument type	*BD LSRFortessa™ (BD Biosciences)*
	Software	*BD FACSDiva software version 8.0.1 (BD Biosciences)*
	Measurement parameters	*See flow data repository of the International Society for Advancement of Cytometry*
**Data Analysis**	Compensation	*Automatic compensation was conducted through FlowJo (Tree Star) prior to importing compensated datafiles into cytobank*
	Gating	*Gating and analysis was performed in cytobank, gating strategy was assessed through backgating of identified populations*

Table S2. Comparison of phosphorylation of ERK, Nf-κB, p38, STAT1 Y701, STAT1 S727, STAT3 Y705, STAT3 S727, STAT4 Y693, STAT5 Y694 (median MdFI and 95% confidence interval of median MdFI) in T cells from pSS patients (n=25*) and healthy donors (n=25**), by time following TLR7 and -9 stimulation of PBMC cultures. * 2 patients not included at 60 and 120 minutes, ** 1 healthy donor not included at 180 and 240 minutes.

Phospho epitope	Time (minutes)	pSS patients		Healthy donors		Comparison
		Median	95% CI*	Median	95% CI*	P**
**NF-κB**	0	148.7	147.3 to 153.5	145.7	141.5 to 148.7	**0.0128**
	15	4.7	3.3 to 6.4	4.0	2.5 to 5.9	0.5601
	30	-4.0	-5.1 to -2.8	-1.6	-2.6 to 0.8	**0.0032**
	60	-1.7	-2.5 to -0.3	-0.8	-3.3 to 2.5	0.5904
	120	0.2	-2.5 to 4.6	3.8	0.8 to 5.4	0.1735
	180	8.0	5.1 to 10.3	11.8	8.7 to 14.4	0.0503
	240	9.4	5.8 to 11.0	15.3	10.7 to 17.3	**0.0040**
**P38**	0	60.1	57.8 to 61.0	56.8	54.9 to 57.8	**0.0016**
	15	-4.0	-4.7 to -2.6	-3.8	-4.3 to -1.9	0.3233
	30	-4.0	-5.6 to -2.4	-3.7	-4.4 to -2.4	0.4048
	60	-2.5	-3.8 to -0.5	-1.7	-4.1 to -0.6	0.9134
	120	7.8	5.3 to 8.9	9.7	7.4 to 11.4	0.0984
	180	9.4	7.3 to 12.0	9.5	7.8 to 12.3	0.8149
	240	7.0	4.6 to 10.4	7.7	4.3 to 11.3	0.5955
**ERK1/2**	0	76.1	74.4 to 79.1	73.4	70.1 to 75.2	**0.0199**
	15	0.0	-1.2 to 1.1	0.7	-0.2 to 1.8	0.1748
	30	-0.3	-1.7 to 0.8	0.2	-0.8 to 1.6	0.2868
	60	-1.8	-2.6 to -1.2	-1.7	-2.8 to -0.2	0.8963
	120	0.8	-0.8 to 2.0	1.0	-0.4 to 1.9	0.6776
	180	1.2	0.1 to 2.2	1.3	-0.6 to 2.4	0.6228
	240	4.1	2.3 to 5.6	2.5	1.1 to 5.2	0.2945
**STAT4 Y693**	0	14.0	13.4 to 14.3	13.1	12.4 to 13.7	0.0539
	15	0.3	0.1 to 0.7	0.4	0.0 to 0.6	0.7391
	30	0.2	0.0 to 0.6	0.4	0.1 to 0.5	0.4157
	60	2.1	1.5 to 3.0	3.1	1.9 to 5.2	0.1429
	120	54.9	40.4 to 64.0	57.6	54.4 to 63.9	0.3536
	180	59.1	51.3 to 66.0	59.3	52.2 to 66.7	0.8302
	240	46.8	42.9 to 53.5	49.6	43.3 to 55.3	0.4538
**STAT5 Y694**	0	107.5	103.6 to 110.2	98.5	96.2 to 101.0	**<0.0001**
	15	-23.7	-25.1 to -21.3	-20.3	-21.9 to -19.7	**0.0042**
	30	-22.9	-26.0 to -20.4	-19.8	-22.3 to -18.6	0.0910
	60	-17.3	-19.8 to -13.0	-13.8	-17.8 to -9.8	0.2162
	120	10.2	3.6 to 16.8	24.3	16.9 to 28.4	**0.0147**
	180	-0.3	-2.0 to 2.0	1.9	-1.3 to 7.0	0.1212
	240	-5.5	-8.4 to -2.0	-3.1	-4.7 to 0.0	0.0946
**STAT1 Y701**	0	118.1	115.2 to 121.6	110.4	104.7 to 113.5	**<0.0001**

	15	-7.8	-9.1 to -6.3	-5.4	-6.6 to -4.0	**0.0040**
	30	-8.5	-10.1 to -6.4	-6.0	-8.2 to -3.8	**0.0070**
	60	8.8	1.4 to 11.9	15.7	5.6 to 24.4	0.0541
	120	143.0	127.8 to 169.7	135.9	114.0 to 154.6	0.5632
	180	117.9	105.2 to 131.4	109.8	99.2 to 130.8	0.4778
	240	92.5	83.1 to 101.6	97.9	86.6 to 109.3	0.3331
**STAT1 S727**	0	31.4	30.5 to 32.0	28.1	27.9 to 29.5	**<0.0001**
	15	-1.3	-1.5 to -0.8	-0.7	-1.3 to -0.5	0.0988
	30	-1.5	-2.1 to -1.2	-0.7	-1.1 to -0.3	**0.0189**
	60	-1.3	-2.0 to -0.4	-0.6	-1.4 to 0.0	0.1266
	120	4.8	3.6 to 7.3	5.9	4.5 to 7.9	0.4827
	180	7.5	6.2 to 8.9	8.5	7.3 to 10.7	0.2349
	240	5.9	4.8 to 6.8	7.6	5.7 to 9.1	**0.0414**
**STAT3 Y705**	0	118.2	112.6 to 128.3	129.7	114.2 to 133.3	0.4269
	15	-4.7	-8.9 to -3.4	-4.1	-7.3 to -2.8	0.5345
	30	-7.1	-10.3 to -5.3	-7.7	-10.8 to -4.8	0.9029
	60	15.8	8.1 to 22.2	17.0	13.3 to 25.4	0.1215
	120	100.4	81.7 to 141.0	124.8	111.0 to 143.3	0.0984
	180	81.0	64.5 to 103.6	98.7	82.1 to 111.7	0.1073
	240	61.6	39.1 to 69.2	68.6	57.5 to 77.0	0.2594
**STAT3 S727**	0	496.5	470.5 to 509.5	476.5	467.0 to 494.2	0.2868
	15	-6.6	-11.1 to -4.2	-2.7	-10.6 to 1.4	0.4731
	30	-13.9	-18.3 to -12.1	-12.8	-16.3 to -6.0	0.3233
	60	7.8	-0.6 to 27.1	14.5	2.6 to 28.3	0.6045
	120	162.4	132.9 to 259.4	236.2	205.5 to 267.4	0.0753
	180	187.8	139.3 to 213	202.3	172.7 to 246.1	0.0553
	240	125.3	82.7 to 138.4	140.1	120.6 to 152.1	0.0608

* 95% CI: 95% confidence interval of median, ** Unpaired Mann-Whitney test.

Table S3. Comparison of phosphorylation of ERK, Nf-κB, p38, STAT1 Y701, STAT1 S727, STAT3 Y705, STAT3 S727, STAT4 Y693, STAT5 Y694 (median MdFI and 95% confidence interval of median MdFI) in B cells from pSS patients (n=25*) and healthy donors (n=25**), by time following TLR7 and -9 stimulation of PBMC cultures. * 2 patients not included at 60 and 120 minutes, ** 1 healthy donor not included at 180 and 240 minutes.

Phospho epitope	Time (minutes)	pSS patients		Healthy donors		Comparison
		Median	95% CI*	Median	95% CI*	P**
**NF-κB**	0	112.7	109.2 to 117.4	115.9	107.1 to 119.0	0.5219
	15	66.7	46.9 to 98.2	55.1	46.5 to 63.9	0.1253
	30	86.2	70.3 to 129.5	77.2	69.3 to 93.6	0.3329
	60	110.7	90.9 to 135.0	103.7	93.7 to 127.5	0.8651
	120	98.7	88.5 to 107.9	97.6	92.1 to 111.1	0.9448
	180	96.2	80.9 to 102.6	98.3	90.5 to 106.6	0.3040
	240	86.1	78.3 to 93.3	94.6	82.4 to 101.3	0.0946
**P38**	0	56.7	55.3 to 58.3	60.5	55.9 to 63.4	0.0910
	15	7.7	4.6 to 11.6	4.3	3.0 to 7.3	0.0873
	30	11.5	7.2 to 14.2	6.6	5.8 to 10.2	0.1159
	60	18.1	11.6 to 20.7	12.0	9.2 to 16.8	0.1873
	120	15.1	14.0 to 20.6	13.8	10.3 to 19.1	0.2843
	180	13.0	12.0 to 14.9	11.0	8.8 to 15.6	0.4778
	240	8.6	6.7 to 13.2	9.1	5.7 to 12.6	0.8144
**ERK1/2**	0	71.2	68.0 to 75.1	73.0	70.5 to 75.1	0.4496
	15	6.5	4.6 to 9.1	7.5	6.5 to 10.1	0.5472
	30	8.5	6.1 to 11.6	9.0	5.7 to 12.9	0.5731
	60	9.2	6.3 to 11.6	9.2	6.1 to 11.9	0.9611
	120	13.2	9.7 to 16.0	13.6	10.6 to 16.2	0.7692
	180	12.0	10.2 to 15.2	11.8	8.8 to 13.4	0.6654
	240	14.3	10.6 to 19.7	13.0	9.6 to 15.5	0.2427
**STAT4 Y693**	0	8.6	8.2 to 9.1	9.6	8.3 to 10.8	0.1405
	15	-0.1	-0.7 to 0.4	-0.1	-0.8 to 0.5	0.8726
	30	-0.3	-0.6 to 0.6	0.0	-0.4 to 0.2	0.9791
	60	-0.4	-1.3 to 0.3	-0.2	-0.8 to 0.2	0.8164
	120	0.1	-0.2 to 0.8	0.0	-1.0 to 1.1	0.8173
	180	0.3	-0.6 to 1.7	0.0	-0.7 to 1.1	0.3531
	240	0.6	-0.1 to 0.8	0.2	-0.3 to 0.8	0.7687
**STAT5 Y694**	0	83.8	82.3 to 87.7	90.7	85.6 to 94.1	0.0539

	15	-19.9	-22.5 to -18.4	-21.0	-24.0 to -18.6	0.8126
	30	-19.6	-24.4 to -17.8	-22.0	-25.2 to -20.0	0.3329
	60	-15.8	-19.5 to -13.3	-18.9	-20.9 to -16.8	0.2089
	120	-6.7	-10.7 to 0.1	-6.0	-8.7 to 2.8	0.4575
	180	4.3	-1.7 to 9.8	10.1	-1.4 to 16.5	0.0729
	240	6.4	2.4 to 9.5	9.3	3.6 to 15.7	0.0729
**STAT1 Y701**	0	96.5	91.6 to 104.3	97.7	89.0 to 101.6	0.8877
	15	-4.6	-8.1 to -2.6	-4.1	-5.8 to -0.8	0.6402
	30	-8.4	-10.7 to -4.9	-4.9	-8.7 to -2.2	0.1353
	60	-1.8	-6.5 to 2.2	5.9	-5.6 to 9.8	0.1319
	120	63.7	54.3 to 82.7	58.2	52.9 to 86.8	0.6635
	180	150.6	129.0 to 169.3	149.3	114.2 to 171.9	0.5815
	240	148.0	135.4 to 172.0	151.9	132.1 to 162.7	0.8144
**STAT1 S727**	0	29.0	28.0 to 30.2	27.6	25.8 to 29.0	0.0646
	15	0.9	0.7 to 2.9	1.1	-0.1 to 1.9	0.2150
	30	5.7	4.1 to 7.4	3.2	1.6 to 3.7	**0.0001**
	60	12.9	8.3 to 17.7	7.2	5.4 to 8.5	**0.0021**
	120	25.2	23.0 to 30.4	18.3	15.0 to 21.9	**0.0003**
	180	45.9	37.9 to 49.6	40.8	34.5 to 45.9	0.2193
	240	39.4	31.3 to 47.2	37.9	30.9 to 46.2	0.8919
**STAT3 Y705**	0	81.24	80.2 to 84.7	83.92	79.7 to 91.0	0.2150
	15	1.6	-0.3 to 4.0	1.6	-2.3 to 2.7	0.3233
	30	0.3	-1.9 to 1.9	0.2	-5.8 to 1.0	0.5094
	60	1.6	-2.0 to 4.0	-0.4	-4.3 to 2.1	0.2015
	120	21.0	15.1 to 25.1	24.1	17.5 to 31.4	0.4575
	180	29.7	22.0 to 32.6	26.8	18.1 to 35.8	0.7687
	240	28.6	24.6 to 32.3	28.2	24.4 to 33.8	0.8298
**STAT3 S727**	0	271.3	267.0 to 292.6	295.1	270.2 to 330.4	0.0873
	15	41.2	22.3 to 49.4	20.0	7.7 to 26.1	**0.0035**
	30	83.9	63.3 to 109.9	59.4	43.5 to 71.8	**0.0080**
	60	138.8	112.0 to 155.0	96.5	77.3 to 127.0	**0.0069**
	120	178.4	155.2 to 196.9	145.2	121.4 to 156.7	**0.0074**
	180	184.0	164.9 to 208.3	168.4	145.0 to 194.5	0.2427
	240	152.8	126.5 to 174.6	156.5	132.7 to 169.3	0.7238

* 95% CI: 95% confidence interval of median, ** Unpaired Mann-Whitney test.

Table S4. Comparison of phosphorylation of ERK, Nf-κB, p38, STAT1 Y701, STAT1 S727, STAT3 Y705, STAT3 S727, STAT4 Y693, STAT5 Y694 (median MdFI and 95% confidence interval of median MdFI) in NK cells from pSS patients (n=25*) and healthy donors (n=25**), by time following TLR7 and -9 stimulation of PBMC cultures . * 2 patients not included at 60 and 120 minutes, ** 1 healthy donor not included at 180 and 240 minutes.

Phospho epitope	Time (minutes)	pSS patients		Healthy donors		Comparison
		Median	95% CI*	Median	95% CI*	P**
**NF-κB**	0	121.4	115.0 to 125.8	120.3	114.8 to 122.2	0.6130
	15	6.7	5.5 to 8.2	6.6	4.8 to 7.4	0.7247
	30	3.4	-3.4 to 6.9	4.2	2.9 to 6.4	0.2010
	60	6.3	2.0 to 10.1	11.9	9.4 to 13.1	**0.0261**
	120	19.2	14.0 to 35.6	44.6	33.6 to 49.0	**0.0003**
	180	12.1	7.0 to 19.9	26.0	15.7 to 31.7	**0.0056**
	240	-15.5	-36.4 to -3.0	6.4	-8.8 to 16.2	**0.0168**
**P38**	0	66.5	64.5 to 71.5	64.6	61.6 to 65.8	**0.0318**
	15	-1.0	-3.1 to 0.6	-2.4	-2.9 to -1.0	0.2452
	30	-1.1	-2.7 to 1.2	-1.2	-2.6 to -0.1	0.5219
	60	-0.9	-2.3 to 1.1	0.3	-1.3 to 2.9	0.5625
	120	13.6	11.6 to 15.9	28.2	17.8 to 32.0	**0.0003**
	180	15.6	11.6 to 19.5	19.8	15.8 to 24.3	**0.0528**
	240	7.9	5.8 to 10.6	11.6	8.2 to 15.7	**0.0580**
**ERK1/2**	0	73.1	69.4 to 75.7	71.7	67.3 to 74.0	0.2223
	15	2.3	-0.3 to 3.5	3.5	1.0 to 4.8	0.1570
	30	1.5	0.4 to 2.9	3.3	0.4 to 4.4	0.2531
	60	2.3	1.5 to 5.2	1.7	0.2 to 2.8	0.2015
	120	5.6	3.8 to 8.9	6.6	3.7 to 8.2	0.7085
	180	5.0	2.3 to 7.9	7.0	3.5 to 11.1	0.0946
	240	11.7	8.2 to 17.2	13.5	8.2 to 18.8	0.8767
**STAT4 Y693**	0	17.6	16.3 to 18.2	16.3	15.9 to 18.1	0.5862

	15	1.4	1.0 to 2.0	1.5	1.1 to 2.0	0.8726
	30	2.0	0.8 to 2.6	0.9	0.6 to 2.0	0.3940
	60	3.8	2.1 to 4.6	7.7	5.2 to 10.4	**0.0021**
	120	62.9	39.2 to 74.2	99.2	76.6 to 103.3	**0.0009**
	180	54.0	40.3 to 68.9	73.0	62.7 to 85.8	**0.0111**
	240	33.2	16.7 to 44.9	43.8	32.8 to 53.6	**0.0234**
**STAT5 Y694**	0	118.7	111.7 to 122.6	109.1	106.2 to 114.7	**0.0026**
	15	-22.7	-24.4 to -19.9	-20.1	-21.2 to -18.7	0.0565
	30	-22.5	-23.6 to -19.5	-18.7	-21.0 to -17.9	0.0737
	60	-19.6	-21.7 to -16.3	-17.4	-22.0 to -14.8	0.5904
	120	-14.2	-20.4 to -11.4	-14.1	-16.6 to -10.7	0.3126
	180	-11.2	-16.2 to -9.3	-13.4	-17.2 to -10.0	0.3230
	240	-10.7	-17.5 to -7.6	-11.1	-14.4 to -7.0	0.9075
**STAT1 Y701**	0	90.0	85.5 to 94.4	83.7	82.5 to 87.0	**0.0169**
	15	-3.2	-4.2 to 0.4	-1.0	-2.5 to 1.7	0.1458
	30	-2.7	-4.6 to -1.3	-0.3	-2.3 to 1.2	**0.0318**
	60	10.6	4.8 to 12.2	18.3	8.9 to 25.5	0.0753
	120	90.4	79.9 to 98.5	72.8	67.5 to 79.3	**0.0361**
	180	63.9	56.6 to 74.2	59.0	52.0 to 64.7	0.2592
	240	41.9	30.6 to 54.1	44.9	38.5 to 54.4	0.3744
**STAT1 S727**	0	30.3	29.6 to 31.6	28.5	27.1 to 29.5	**0.0070**
	15	-0.3	-1.3 to 0.6	-0.4	-0.8 to 0.7	0.3329
	30	-1.0	-1.9 to 0.0	-0.5	-1.5 to 0.1	0.1353
	60	-1.9	-2.4 to -1.3	-0.9	-1.7 to -0.2	**0.0380**
	120	4.5	2.8 to 5.8	4.4	2.7 to 5.5	0.9945
	180	4.4	2.3 to 6.0	4.2	3.3 to 5.6	0.6948
	240	1.3	-2.5 to 2.8	2.5	-0.8 to 3.4	0.1776
**STAT3 Y705**	0	97.8	92.6 to 100.3	94.9	91.6 to 97.8	0.1811
	15	-0.1	-3.6 to 2.3	0.4	-1.1 to 4.1	0.2781
	30	1.3	-1.5 to 5.5	0.9	-0.9 to 3.3	0.2868
	60	0.0	-2.6 to 2.6	1.0	-2.2 to 4.1	0.3754
	120	26.8	19.4 to 33.1	27.0	21.4 to 32.0	0.8651
	180	12.7	8.6 to 19.5	14.9	8.0 to 16.0	0.8302
	240	9.1	4.8 to 15.8	10.3	5.2 to 13.2	0.8452
**STAT3 S727**	0	257.4	241.3 to 298.4	275.3	260.1 to 295.1	0.1458
	15	-8.4	-13.7 to 0.2	-12.6	-15.0 to -7.9	0.2374
	30	-10.1	-13.7 to -2.4	-8.7	-11.2 to -6.8	0.8425
	60	-0.9	-6.4 to 1.0	-2.7	-6.9 to 5.9	0.8642
	120	60.8	39.7 to 75.8	90.4	66.4 to 108.5	**0.0026**
	180	40.6	22.8 to 57.7	57.3	38.8 to 91.2	**0.0275**
	240	-30.6	-129.2 to 7.4	14.2	-13.5 to 44.7	0.0580

* 95% CI: 95% confidence interval of median, ** Unpaired Mann-Whitney test.

Table S5. Comparison of plasma cytokine levels (median pg/ml and 95% confidence interval of median) showing significant differences between pSS patients (n=25) (except IL-15, n= 21; and IL-4, n= 22) and healthy donors (n=25) (except IL-6, n=24; TNF-α, n= 23; and IL-4, n= 16).

**Cytokine**	**pSS patients**		**Healthy donors**		**Comparison**
	**Median**	**95% CI***	**Median**	**95% CI***	**P****
**IL-1β**	18.96	18.96 to 85.66	18.96	18.96 to 18.96	**0.0004**
**IL-13**	16	16 to 58.78	16	16 to 16	0.0207
**IL-6**	0.3254	0.3254 to 10.18	0.3254	0.3254 to 0.3254	**0.0043**
**IL-12**	200	200 to 288.2	140.4	110 to 140.4	<0.0001
**MIP-1α**	84.45	84.45 to 183.3	56.13	56.13 to 56.13	<0.0001
**MIP-1β**	66.05	46.95 to 156.6	46.95	46.95 to 46.95	0.0008
**MCP-1**	293.5	256.6 to 401.6	219.2	181.1 to 256.6	0.0029
**IL-15**	113.7	21.51 to 194.5	21.51	21.51 to 21.51	0.0001
**IFN-α**	139	88.51 to 139	88.51	88.51 to 88.51	<0.0001
**TNF-α**	7.758	7.758 to 45.95	7.758	7.758 to 7.758	0.0027
**IL-2**	6.368	6.368 to 20.93	6.368	2 to 6.368	<0.0001
**IL-4**	36.8	36.8 to 270.2	36.8	36.8 to 36.8	0.0049

* 95% CI: 95% confidence interval of median, ** Unpaired Mann-Whitney test.

Table S6. Comparison of plasma cytokine levels (median pg/ml and 95% confidence interval of median) showing significant differences between SSA+ pSS patients (n=12) and SSA- pSS patients (n=13), (except for IL-4, n=11 in each category).

Cytokine	SSA+ patients		SSA- patients		Comparison
	**Median**	**95% CI***	**Median**	**95% CI***	**P****
**IL-1β**	108.1	18.96 to 182.6	18.96	18.96 to 34.35	**0.0199**
**MCP-1**	401	293.5 to 1337	256.6	219.2 to 293.5	0.0292
**IFN-α**	160	88.51 to 423	88.51	88.51 to 139	**0.0214**
**IL-2**	20.93	6.368 to 49.45	6.368	6.368 to 6.368	0.0049
**IL-4**	228.9	36.8 to 889	36.8	36.8 to 94.19	0.0339

* 95% CI: 95% confidence interval of median, ** Unpaired Mann-Whitney test.
